# What Intervention Techniques Are Effective in Changing Positive Affective Variables and Physical Activity? A Systematic Review and Meta-Analysis

**DOI:** 10.3389/fpsyg.2021.628993

**Published:** 2021-06-10

**Authors:** Cheng Chen, Emily Finne, Alexandra Kopp, Darko Jekauc

**Affiliations:** ^1^Institute of Sports and Sports Science, Karlsruhe Institute of Technology (KIT), Karlsruhe, Germany; ^2^Department Prevention and Health Promotion, School of Public Health, Bielefeld University, Bielefeld, Germany; ^3^Department of Sport Science, Institute of Sport Sciences, Humboldt University of Berlin, Berlin, Germany

**Keywords:** intervention, technique, positive affective variable, physical activity, moderator

## Abstract

A recent meta-analysis has demonstrated that positive affective variables (PAVs) partially mediate physical activity (PA) interventions. However, the effectiveness of each intervention technique on PAVs and PA is still unknown. Thus, this meta-analytic review included two primary objectives: (1) to summarize intervention effects on PA and PAVs; (2) to examine each behavior change technique's effectiveness in modifying PAVs and PA. Following PRISMA protocols, we had searched five electronic databases by April 1, 2020. The random-effect model in the Comprehensive Meta-Analysis Version 3 was adopted to perform these meta-analytic analyses. The search identified 1,742 articles, and 37 studies (49 datasets) met our inclusion criteria. Finally, inferential statistics yielded that: the utilization of “teach to use prompts/cues,” “facilitate social comparison,” and “provide information on consequences of behavior in general” had positive effects on PA or PAVs outcomes; the utilization of “barrier identification/problem solving” and “plan social support/social change” negatively affected on PA or PAVs outcomes. However, there was considerable heterogeneity in the findings. Nonetheless, this paper has considerable implications for guiding future comparative intervention studies to achieve more reliable outcomes.

## Introduction

Regular physical activity (PA) is highly beneficial for the prevention of premature mortality (Ekelund et al., 2016) and for physical and mental health (Penedo and Dahn, 2005). However, only a minority of modern adults report that their PA participation levels align with most public health guidelines. Besides, a further 50% of exercisers drop out within the first 6 months after initial participation (Finne et al., 2019). Exploring ways to promote and maintain PA is necessary because the benefits are not sustainable without consistent and regular attendance (Annesi, 2003).

### The Unfavorable Commonality in Mainstream Physical Activity Change Theories

Current mainstream theoretical approaches used for PA interventions include social cognitive theory (SCT; Bandura, 1998), the theory of planned behavior (TPB; Ajzen, 1991), the trans-theoretical model (TPB; Prochaska and Velicer, 1997), and self-determination theory (SDT; Deci et al., 1994). According to SCT, PA variations are regulated by reciprocal determinations among personal cognitive factors (e.g., self-efficacy, outcome expectations, knowledge), the physical and social environment (e.g., observational learning, normative beliefs, social support, opportunities, and barriers), and behavioral factors (e.g., behavioral skills, intentions, reinforcement) (Bandura, 2004). TPB comprises three core components, namely, attitude, subjective norms, and perceived behavioral control, which together shape individuals' PA intentions and behavior (Ajzen, 1991). The TTM has concentrated on stages of change, processes of change, levels of change, self-efficacy, and temptation (Prochaska and Velicer, 1997). And SDT emphasizes the role of autonomy, competence, and relatedness for PA interventions (Deci and Ryan, 2008). All of these theories share a core attribute that stems from cognitivism. In detail (1) they all emphasize the primacy of imagined end states (behaviors or goals) (Brand and Ekkekakis, 2018) in PA change, and (2) affective constructs are either entirely omitted or subordinated to cognitive devices, while the idea that affective constructs can serve as motivational forces outside of cognitivism (e.g., momentary emotions associated with physical activity situations; Ekkekakis, 2017) is ignored. Consistent with these theories, PA interventions have focused primarily on techniques that provide education about PA's benefits, build perceived ability, and self-regulation to perform PA (Conn et al., 2011; Chase, 2015; Rhodes et al., 2019). However, even as the framework predicting the highest amount of PA variance, the social cognitive theory can only explain, on average, 20% of the variation in PA maintenance (Jekauc et al., 2015). Rhodes et al. (2009a) integrated 34 PA intervention studies, and found that 85% of the findings showed that affective expectations were notable predictors of PA behavior (*r* = 0.43; 95% CI = 0.36–0.46), whereas only 35% of the findings showed that instrumental expectations were significant predictors of PA behavior (*r* = 0.25; 95% CI = 0.21–0.29). Hence, perhaps the failure to separate the affective and instrumental reflections or expectations of the intervention on PA hinders the exploration process of PA promotion (McEwan et al., 2016; Jekauc and Brand, 2017). Therefore, a more refined meta-analysis dedicated to affective variables and PA is inevitable (Jekauc et al., 2015).

### Definitions of Positive Affective Variables

In general, “affect is the experiential state of feeling and is a collective term describing feeling states such as emotion and mood” (Gellman and Turner, 2013). Affective states may vary in several aspects, such as their duration, intensity, specificity, pleasantness, and degree of arousal, and they have essential roles in regulating cognition, behavior, and social interaction. As a superordinate category, emotions and moods belong to affect. Emotions and moods differ mainly in (1) their duration: emotions are rather brief and intense experiences, and moods last somewhat longer than emotions, and (2) whether they are directed to a specific cause: emotions are reactions to specific external stimuli (i.e., objects or events) and may arise relatively automatically or after a cognitive assessment of the stimulus; moods are more diffuse in nature (Gellman and Turner, 2013). Furthermore, the concept of attitude is considered to represent relatively enduring beliefs and preferences for a particular organism and is primarily composed of cognitive, affective, and motivational components (Breckler, 1984). Contrary to the caution of theorists, namely that these concepts should be distinguished, affect, emotion, feeling, mood and affective attitude (Ostrom, 1969) are often used liberally in empirical researches (Batson et al., 1992). Thus, this paper integrated them into a generalized term as affective variables. Besides, some other theorists have noted that organizing affective variables by dimension may be more meaningful than considering them by category (Cacioppo and Gardner, 1999; Watson et al., 1999; Shiota and Kalat, 2012). Thus, we generalized non-negative affect, emotion, feeling, mood, and affective attitude and use the term “positive affective variables (PAVs)” to refer to them.

### Approach-Avoidance Distinction of Affective Variables and Motivation in Physical Activity

According to existing research, the approach-avoidance distinction is applicable in affective variables (positive and negative affective dispositions) (Watson et al., 1999). The neurological underpinnings have also given evidence of this linkability between motivation and emotion through affective neuroscience (Davidson, 2003). As Larsen et al. (2008) stated, “motivation and valence tend to be correlated, such that positive emotions are associated with approach and negative emotions with avoidance.” Consistently, it could also be shown that positive emotions (e.g., enjoyment) during PA and intrinsic motivation for PA possibly share common determinants (Wienke and Jekauc, 2016). Furthermore, several other theoretical and empirical studies also have shown that PAVs are essential determinants of PA behavior or outcomes (Rhodes et al., 2009b). Following the upward spiral theory of lifestyle change, motivation is significantly associated with positive affects experienced during healthy behaviors, and motivational salience subconsciously guides attention to these behaviors and decisions to repeat them (van Cappellen et al., 2018). Further, a recent meta-analysis emphasized that the PAV emerges as a significant mediator between intervention and PA outcomes (Chen et al., 2020). Based on these neuroscientific, theoretical, and empirical fundamentals, enhancing PAVs is more likely to facilitate physical activity than activities that rely primarily on extrinsic motivation, such as those expected to improve health and well-being (Nielsen et al., 2014).

### Empirical Studies on Positive Affective Variables and Physical Activity

In recent years, there has been an upsurge of enthusiasm to consider the role of PAVs in PA prescribing more (e.g., Ekkekakis et al., 2013, 2020), but our knowledge of how to change PAVs and subsequent PA remains deficient. So far, Rhodes and his colleagues have conducted three reviews (Rhodes et al., 2009a, 2019; Rhodes and Kates, 2015), which summarized the relationship between affective response/affective judgment (i.e., thoughts about the overall pleasure/displeasure, enjoyment, and feeling states expected from enacting a behavior) and PA. Initially, through 82 correlational studies and 20 eligible experimental studies, Rhodes et al. (2009a) demonstrated a medium-effect size relationship between affective judgment and PA. A significant positive correlation between affective judgments and PA was reported in 83 out of 85 correlational samples, with a pooled r of 0.42 (95% CI = 0.37–0.46). A further meta-analytic synthesis was reported in 2015. It stated that positive changes in primary affective responses during moderate-intensity exercise were associated with future PA intention (Rhodes and Kates, 2015). A recent review explored interventions to manipulate adults' (of healthy and unhealthy populations) affective judgments and subsequent PA, but no technique was considered adequate (Rhodes et al., 2019). We speculate that two main reasons influenced these results. First, it is well-known that many diseases (e.g., Alzheimer's disease) can change emotional regulation (Bucks and Radford, 2004), so it is necessary to distinguish between healthy and unhealthy populations. Second, we presumed that Rhodes et al. (2019) did not distinguish between intrinsic motivation and affect in the literature inclusion (Silva et al., 2010a,b; Moustaka et al., 2012; Kinnafick et al., 2016; Shah et al., 2016) leading to these outcomes. According to Weinberg and Gould (2014, pp. 139), intrinsic motivation includes knowledge, accomplishment, and stimulation, while affect is merely a part of intrinsic motivation (stimulation). Besides, we desired to exclude negative affective variables from this study (Egloff, 1998; Reich et al., 2001). The reasons were: (1) Reich et al. (2001) conducted two experiments based on the two-factor model and the bipolar model, which showed that the cognitively more complex participants reported the mutual independence of positive and negative affect, while those with simpler cognitions reported the polarity of positive and negative affect, which meant that positive and negative affects could be differentiated for exploration; (2) as we described in the previous paragraph, the approach-avoidance distinction was also applicable in the affective variables; (3) Chen et al. (2020) distinguished between positive and negative affective variables and demonstrated the significant mediating role of PAVs in the PA intervention. Overall, we would like to implement a more nuanced meta-analysis to understand how PAVs and PA can be manipulated in healthy populations.

Considering the aforesaid, this paper included two primary objectives. First, to summarize intervention effects on PA and PAVs; second, examine each behavior change technique's effectiveness in modifying PAVs and PA and exploring potential demographic and methodological moderators. That is, we investigated (1) which methodological factors moderated the outcomes of PAVs and PA (e.g., study design, theory framework, intervention duration, measurement, number of intervention techniques used); (2) which demographic characteristics moderated the results of PAVs and PA interventions (e.g., age, gender, population setting, PA level at baseline); (3) which behavior change techniques (BCTs) were the most effective for PAVs and PA interventions.

## Methods

### Search Strategy and Inclusion Criteria

**Figure 1:**
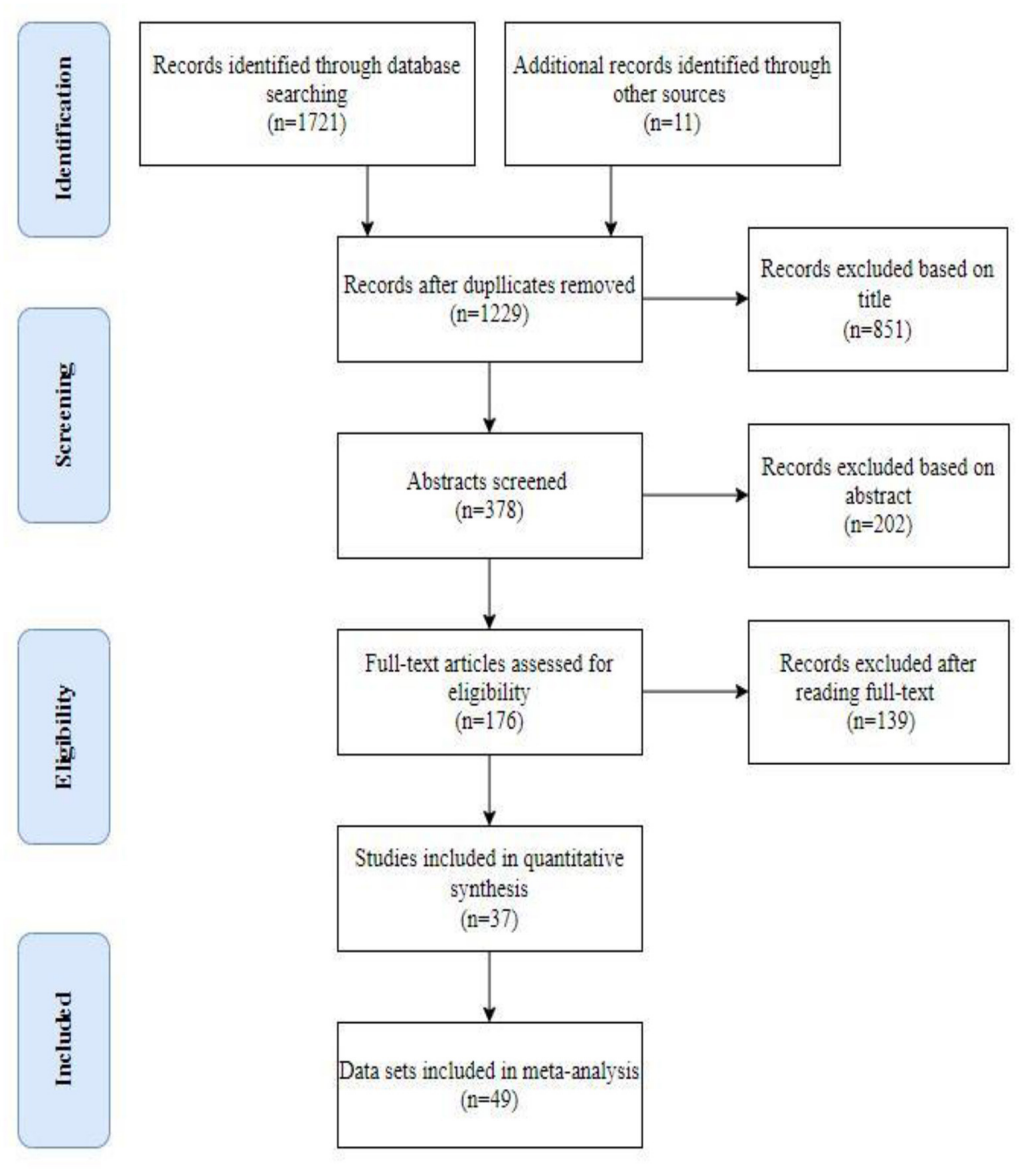
PRISMA flow diagram for articles identified, screened eligible, and included in this paper.

The literature search was conducted according to the PRISMA standard protocol (Moher et al., 2009) (see Figure 1). A structured electronic search strategy was used to retrieve studies published by April 1, 2020. The databases searched included Web of Science, PubMed, PsycINFO, PsycArticle, and Psychology and Behavioral Sciences Collection. The search terms were: (1) Intervention OR Trial OR Experiment; (2) Physical Activity OR Exercise; (3) Enjoy^*^ OR Affect^*^ OR Emotion^*^ OR Mood^*^ OR Feeling; (4) Mechanism^*^ OR Mediat^*^ OR Predict^*^ OR Process^*^ OR “Structural equation modeling” OR Caus^*^ OR Path^*^ OR Correlat^*^ OR Relationship OR Associat^*^; (5) NOT (Patient^*^ OR Cancer OR Clinical OR Disease^*^ OR Illness OR Depression OR Rat OR Mouse OR Protocol OR Cell OR Bone^*^ OR Blood OR Rehabilitation OR Disorder^*^ Injur^*^ OR HIV OR Carbohydrate OR Athlete^*^ OR Player^*^ OR Runner^*^ OR Review OR Comment OR Therapy); (6) 1 AND 2 AND 3 AND 4 AND 5. Besides, more than 98 percent of the search results were in English, and very few studies were published in other languages. Hence, we only included studies published in English for the accuracy of data extraction.

The first author completed the search, and the eligibility of each study was determined by the Cochrane handbook for systematic reviews of intervention studies (Higgins et al., 2019b). Studies in which the first author was unsure whether to be included were discussed and determined with the last author. A study was eligible for our meta-analysis if it met the following criteria: (1) experimental studies assessing PAV as a dependent variable; (2) PAV was a target of the intervention; (3) studies whose goal was to increase lifestyle or recreational PA, not for competitive sports (Caspersen et al., 1985; Vanhees et al., 2005); (4) sufficient data to calculate the effect sizes (Hedges' g) of PAVs and PA; (5) participants were healthy individuals (not a clinically defined population and not pregnant). Furthermore, we intentionally chose a minimum duration of PA of 10 min, given that 10 min is the recommended minimum duration of exercise to elicit health benefits (Edwards and Loprinzi, 2019).

### Data Extraction and Data Analysis

First, the risk of bias assessment was administered using the STROBE standard tool (Elm et al., 2007). The tool includes questions in a “yes” (1) or “no” (0) format (e.g., did the study report the sources and details of PA assessment; did the instruments have acceptable reliability for the specific age group?). Study qualities were assessed by the first and last authors separately, and any differences were resolved through discussion. The studies' quality was then graded as low (scores 0–2), medium (scores 3–4), or high (scores 5–6).

Next, with the supervision and guidance of the last author, the first author completed the extraction of the following data: BCTs; the PAVs' constructs, dimensions, and measurements; PA assessment methods, variables, measures; methodological data (e.g., study design, theory framework, intervention duration, measure employed, number of intervention methods used, primary intervention targeted, PA focus); demographic data (e.g., age, gender, population setting, PA level at baseline). Data for BCTs were extracted based on the 40-item taxonomy by Michie et al. (2011). Coyne et al. (2010) pointed out that several small sample studies can be included in a meta-analysis, but if a meta-analysis includes many small sample studies, it may result in a large bias in its effect size. For this reason, we classified each trial according to whether its sample size was >35 (Kraemer et al., 1998; Coyne et al., 2010) and calculated the sample size as a moderator variable in the calculation.

Finally, we adopted the statistical procedure utilized by Ashford et al. (2010) and Williams and French (2011). The random-effect model in the Comprehensive Meta-Analysis Version 3 (Borenstein et al., 2014) was employed in the calculation. Based on the raw data, we employed Hedges' g to estimate effect sizes (i.e., the adjusted standardized mean difference for both PAVs data and PA data between post-test means in intervention and control group where possible, or pre and post-test means of the intervention group) (Durlak, 2009). With multiple measurement time points, we chose the first measurement taken at the end of the intervention (Higgins et al., 2019b) because those results could maximally be influenced by different interventions and less influenced by other factors relative to the follow-up measurements. To overcome the potential unit-of-analysis error due to the inclusion of multi-arm studies, several approaches have been proposed by Higgins et al. (2019a). Specifically, when exploring the moderating effects of each methodological and demographic variable, we combined all intervention groups within a study to create a single pair-wise comparison (Higgins and Li, 2019). We then computed the summary effect for this intervention group vs. the control group. However, when performing moderator analyses for BCTs, we included each pair-wise comparison separately, but shared control groups were divided into several smaller groups for the different comparisons to avoid “double counting.” Moderator analyses were limited to categories with at least three studies. The findings' heterogeneity was examined using the Q-statistic (Higgins et al., 2003; Hedges and Pigott, 2004); a 5% cut-off was used for significance. The Q coefficient's significance represents the heterogeneity of the dataset beyond what would be expected from sampling error alone, suggesting that additional systematic factors contribute to the variance. Therefore, we performed moderator analyses to explore the causes of heterogeneity by comparing the mean variability of effect size estimates for two groups of studies characterized by the presence or absence of a specific study characteristic (e.g., a specific BCT) (Ashford et al., 2010). Finally, we explored publication bias using Egger's regression intercept (i.e., a statistical test result for funnel plot asymmetry), and a 5% cut-off was used for significance.

## Results

### Study Flow and Characteristics

The search identified 1,732 articles, of which 1,352 were duplicates or could be excluded based on the titles. Of the remaining 389 articles, there were 183 articles for full-text review, of which 11 were identified by cross-referencing (see Figure 1). Finally, 37 studies met our inclusion criteria (see Appendix 8), of which ten studies contained two or three subgroups (Focht et al., 2007; Rose and Parfitt, 2007; Schneider and Cooper, 2011; Fitzsimons et al., 2012; Kraft et al., 2015; Wang et al., 2015; Niedermeier et al., 2017; Noradechanunt et al., 2017; Miragall et al., 2018; Gråstén and Yli-Piipari, 2019). Due to the nature of the data to be analyzed, we included each pair-wise comparison separately when investigating BCTs' moderating effects on PAVs and PA and therefore included a total of 49 data sets. In case of multiple comparisons to the same reference group, we split the control group as described above. Meanwhile, the quality of each study is presented in Appendix 1. Of the 37 studies, six were rated as high quality, 17 were rated as low quality, and the remaining 20 studies were rated as moderate quality.

Furthermore, the general characteristics of the 37 studies are presented in Appendix 2. Then, **Table 2** presents the overall study characteristics of the 37 studies. Concerning the age of the participants, four age intervals were designed to classify the mean age of each study: under 18 years (*n* = 15), 18–35 years (*n* = 13), 36–60 years (*n* = 7), and over 60 years (*n* = 2). In terms of gender distribution, only one group identified its gender as male, 11 studies defined its gender as female, and the remaining 25 studies were mixed gender. For baseline PA, we marked out four classifications as “not meeting PA guidelines at baseline” (*n* = 17), “meeting PA guideline at baseline” (*n* = 3), “mixed” (*n* = 9), and “unreported” (*n* = 8). 33 of the 37 studies stated their theoretical underpinnings, while the other four did not. In addition, 16 interventions were implemented based on multiple theoretical frameworks, and 17 interventions were based on a single theoretical framework (TTM *n* = 3, SCT *n* = 3, TPB *n* = 3, SDT *n* = 3, the dual-mode model *n* = 1, challenge point theory *n* = 1, tactical games model *n* = 1, affective reflective theory *n* = 1, the health promotion model *n* = 1). The intervention duration of the included individual groups ranged from <3 h−4 years, but the majority was between 2 and 6 months (*n* = 12). Only 13 of the 37 studies randomized their subjects. Furthermore, over 55 percent of the interventions were performed in schools, colleges, and university laboratories.

### Contents of BCTs

The intervention techniques employed by each intervention group are summarized in detail in Appendix 5. According to Michie et al. (2011), the 49 independent intervention groups used 2–17 intervention techniques, of which seven interventions employed no more than three intervention techniques, 23 interventions employed 4–10 intervention techniques, and 20 studies employed more than 10 intervention techniques. Further, Table 1 presents the frequency of use of each intervention technique across all included studies. The most frequently used intervention techniques were (1) provide instruction on how to perform the behavior (83.67%), (2) provide instruction on when and where to perform the behavior (81.63%); (3) action planning (57.14%), and model/demonstrate the behavior (57.14%). Six intervention techniques were not employed by any of the included studies: (1) shaping; (2) prompt generalization of a target behavior; (3) prompt identification as a role model/position advocate; (4) prompt anticipated regret; (5) fear arousal; (6) stimulate anticipation of future rewards. Five other intervention techniques were rarely used: (1) provide information on consequences of behavior to individual (4.08%); (2) prompting focus on past success (4.08%); (3) agree behavioral contract (4.08%); (4) provide rewards contingent on effort or progress toward behavior (2.04%); (5) provide rewards contingent on successful behavior (2.04%).

**Table 1:** Frequencies of intervention techniques that were used in the intervention groups in meta-analytic analyses.

**Techniques**		**Number of intervention groups (maximum 49)**	**Percentages (%)**
1	Provide information on consequences of behavior in general	15	30.61
2	Provide information on consequences of behavior to individual	2	4.08
3	Provide information about others' approval	9	18.37
4	Provide normative information about others' behavior	5	10.20
5	Goal setting (behavior)	19	38.78
6	Goal setting (outcome)	6	12.24
7	Action planning	28	57.14
8	Barrier identification/problem solving	18	36.73
9	Set graded tasks	10	20.41
10	Prompt review of behavioral goals	13	26.53
11	Prompt review of outcome goals	3	6.12
12	Provide rewards contingent on effort or progress toward behavior	1	2.04
13	Provide rewards contingent on successful behavior	1	2.04
14	Shaping	0	0
15	Prompt generalization of a target behavior	0	0
16	Prompt self-monitoring of behavior	24	48.98
17	Prompt self-monitoring of behavioral outcome	4	8.16
18	Prompting focus on past success	2	4.08
19	Provide feedback on performance	20	40.82
20	Provide instruction on when and where to perform the behavior	40	81.63
21	Provide instruction on how to perform the behavior	41	83.67
22	Model/demonstrate the behavior	28	57.14
23	Teach to use prompts/cues	6	12.24
24	Environmental restructuring	15	30.61
25	Agree behavioral contract	2	4.08
26	Prompt practice	7	14.29
27	Use of follow-up prompts	3	6.12
28	Facilitate social comparison	4	8.16
29	Plan social support/social change	25	51.02
30	Prompt identification as a role model/position advocate	0	0
31	Prompt anticipated regret	0	0
32	Fear arousal	0	0
33	Prompt self-talk	4	8.16
34	Prompt use of imagery	9	18.37
35	Relapse prevention/coping planning	3	6.12
36	Stress management/ emotional training	24	48.98
37	Motivational interviewing	6	12.24
38	Time management	4	8.16
39	General communication skills training	5	10.20
40	Stimulate anticipation of future rewards	0	0

### Characteristics of PAVs

Appendix 3, Table 2 presents the constructs, dimensions, and measurements of each study's PAVs. First, PAVs could be categorized into two broad constructs of affects and emotional states (Shouse, 2005), with several studies measuring both of them. The dimensions of affect included “affective valence” and “positive affect,” and the measurement methods were “feeling scale (FS)” and “positive and negative scale (PANAS).” Emotional states were further categorized as “enjoyment,” “pleasure,” “exercise-induced feeling,” “affective attitude,” and “mood state.” There were various dimensions and methods to measure emotional states, and the most frequently measured dimension was “enjoyment.” Still, there were also “remembered pleasure,” “revitalization,” “positive engagement,” “vigor,” “activation,” and “excitement.” Accordingly, there were various scales for measuring emotional states, for example, “the physical activity enjoyment scale (PACES),” “the PE enjoyment scale (PEES),” “visual analog scale (VAS) of enjoyment/remembered pleasure,” “the interest/enjoyment subscale of intrinsic motivation inventory (IMI),” “single-item enjoyment scale (SES),” “exercise-induced feeling inventory (EFI),” “semantic differential scale of affective attitude (SD),” “profile of mood states (POMS)” and “mood survey scale (MSS).”

**Table 2:** Overall study characteristics of 37 studies.

**Characteristics**	***N* of intervention groups (maximum 37)**	**Percentages (%)**
**Age**		
<18	15	40.54
18–35	13	35.14
36–50	7	18.92
50–75	2	5.41
**Gender**		
Male	1	2.7
Female	11	29.73
Mixed	25	67.57
**Sample size**		
<35 participants per condition	10	27.03
≥35 participants per condition	27	72.97
**Intervention duration**		
≤ 3 h	6	16.22
3 h−2 months	11	29.73
2–6 months (including 2 months)	12	32.43
>6 months	8	21.62
**Number of intervention methods used**		
1–3 methods used	3	8.11
4–10 methods used	17	45.95
4–11 methods used	17	45.95
**Setting**		
School	12	32.43
University	4	10.81
Lab	4	10.81
Community	5	13.51
Other	12	32.43
**The physical activity level at baseline**		
Not meeting guideline	17	45.95
Meeting guideline	3	8.11
Mixed	9	24.32
Unreported	8	21.62
**Positive affective variables measure**		
Affect	6	16.22
Emotional state	24	64.86
Affect and emotional state	7	18.92
**Positive affective variables_measurements**		
The physical activity enjoyment scale	13	35.14
The positive and negative affect schedule	4	10.81
Feeling scale	3	8.11
IMI	2	5.41
Semantic differential scales of affective attitude	2	5.41
Affective attitude Likert scale	1	2.70
Profile of mood states	1	2.70
Single-item enjoyment scale	1	2.70
The PE enjoyment scale	1	2.70
VAS	1	2.70
Multiple	8	21.62
**Physical activity measure**		
Moderate-vigorous physical activity (objective)	2	5.41
Moderate-vigorous physical activity (subjective)	9	24.32
Steps	4	10.81
Frequency	6	16.22
Intensity	6	16.22
Multiple	10	27.03
**Physical activity measurements**		
Equipment usage log/attendance list	5	13.51
HR monitoring	5	13.51
Pedometer	4	10.81
Accelerometer	3	8.11
2/3/7 day physical activity recall	3	8.11
International physical activity questionnaire	2	5.41
Leisure-time exercise questionnaire	2	5.41
Other questionaires	8	21.62
Multiple	4	10.81
Not reported	1	2.70
**Theory**		
No framework explicitly mentioned	4	10.81
Social cognitive theory	3	8.11
The transtheoretical model	3	8.11
Theory of planned behavior	3	8.11
Self-determination theory	3	8.11
Multiple	16	43.24
Others	5	13.51
**Study design**		
Randomized controlled study	13	35.14
Quasi-experimental study	24	64.86
**Study quality rating**		
Low (1–2)	7	18.92
Medium (3–4)	24	64.86
High (5–6)	6	16.22

*IMI, the interest/enjoyment subscale of intrinsic motivation inventory; VAS, visual analog scale of enjoyment/ remembered pleasure*.

### Characteristics of PA

In general, there were two broad categories of PA measurements: objective and subjective. Appendix 4, Table 2 shows that objective and subjective measures were about equally divided. The primary four objective measurements were the recording list (equipment usage log/attendance list), pedometer, accelerometer, and heart monitor. In contrast, subjective measures of PA were diverse. For example, 7-day physical activity recall (7DPAR), 3-day physical activity recall (3DPAR), the short-form of the international physical activity questionnaire (IPAQ), physical activity time-consuming questionnaire (PATCQ), the children's leisure activities study survey (CLASS), 6-point exercise frequency scale (EFS). The PA variables measured by the studies were also highly diverse, for example, moderate to vigorous physical activity (MVPA), leisure-time physical activity (LTPA), the metabolic equivalent of task (MET), exercise adherence, equipment usage, %Max HR.

### Moderating Effect of Methodological and Demographics Variables on PAVs and PA

**Figure 2:**
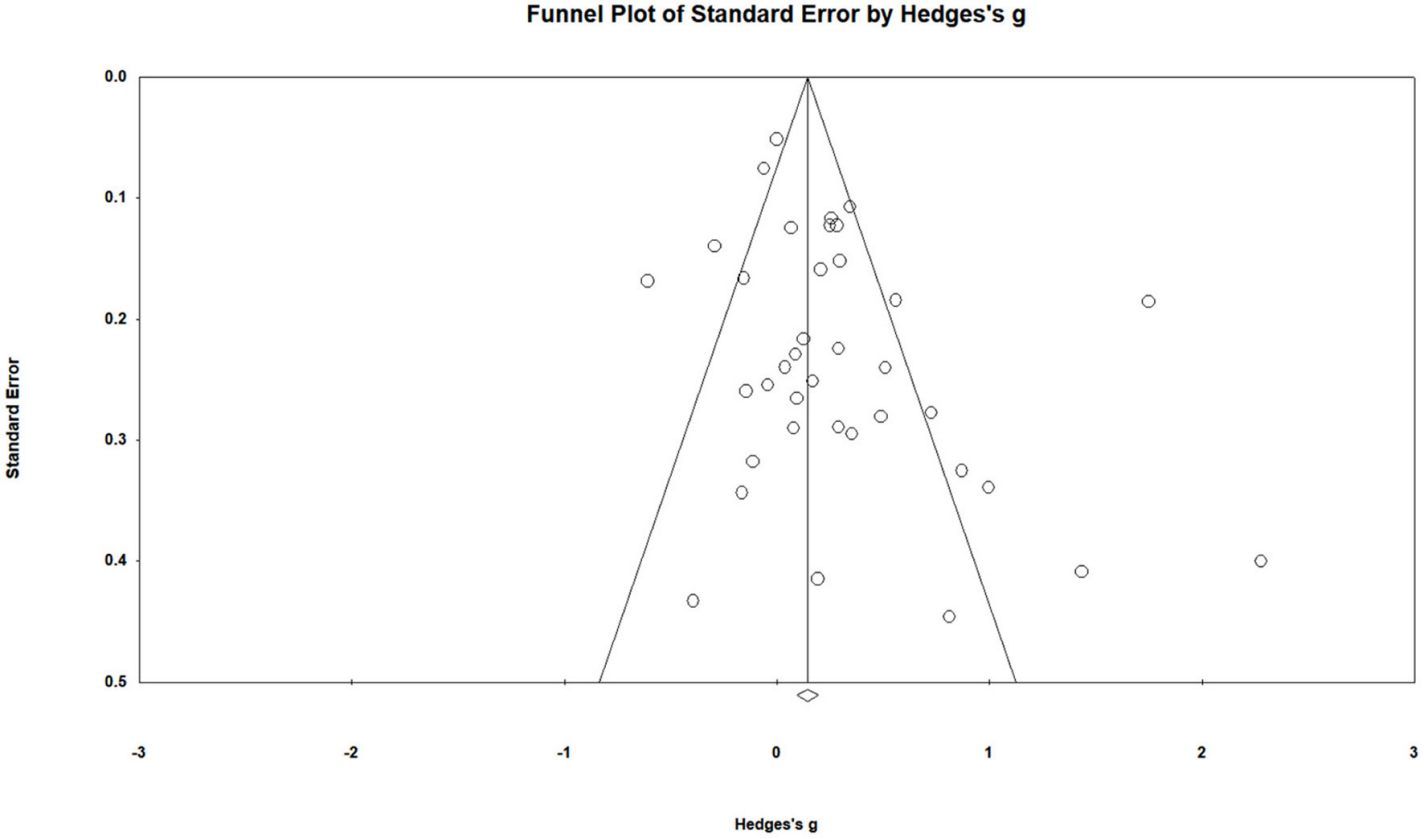
Funnel plot of positive affective variables in this review.

Meta-analytic moderation results of the 37 studies can be found in Table 3. We first reported the moderating effects of demographic and methodological factors on PAVs. Experimental manipulations of PAVs had an overall effect size *g* = 0.28 (95% CI = 0.14–0.41) on PAVs (see Appendix 6). The examination of publication bias for the 37 studies was significant [Egger's intercept *t* = 1.65 (35), *p* = 0.02] (see Figure 2), and in cases such as this with small samples and large heterogeneity, caution should be exercised (Carter et al., 2019). Using the *n* = 35 criterion proposed by Coyne et al. (2010), small-sample bias was a significant moderator in the PAV (*Q* = 6.64; *p* = 0.01) context, with larger effect size (*g* = 0.32, 95% CI = 0.08–0.57) reported for small sample sizes. Age was also a significant moderator to the findings (*Q* = 12.73, *p* < 0.05), mean age interval located between 36 and 50 years reported the largest effect size (*g* = 0.48, 95% CI = 0.12–0.84). There was also a significant moderating effect of gender on PAVs, with the largest effect size for mixed-gender studies (*g* = 0.30, 95% CI = 0.12–0.48). Similarly, there was a significant moderating effect of intervention duration on PAVs, with the largest effect size for intervention duration between 2 h and 2 months (*g* = 0.69, 95% CI = 0.07–1.31). The theory was also a significant moderator in PAVs intervention, with SDT having the largest effect size (*g* = 0.80, 95% CI = 0.33–1.27). However, neither the intervention setting (*Q* = 5.83, *p* = 0.21) nor the baseline level of PA (*Q* = 6.54, *p* = 0.09) were significant moderators in our PAVs investigation.

**Table 3:** Demography and methodology effects of experimental effects on PAVs and PA.

	**PAVs**							**PA**						
	***k***	***g***	**SE**	**95% CI**		***Q***	***p***	***k***	***g***	**SE**	**95% CI**		***Q***	***p***
Point estimate	37	0.28	0.07	0.14	0.41	202.89	0	37	0.30	0.10	0.11	0.48	412.08	0
Age						12.73	<0.05						19.23	<0.01
<18	15	0.14	0.11	−0.09	0.36			15	0.36	0.17	0.03	0.69		
18–35	13	0.31	0.10	0.11	0.51			13	0.44	0.27	−0.10	0.97		
36–50	7	0.48	0.18	0.12	0.84			7	0.37	0.09	0.18	0.55		
Gender						14.33	<0.01						11.53	<0.01
Female	11	0.07	0.06	−0.05	0.19			11	−0.08	0.15	−0.39	0.22		
Mixed	25	0.30	0.09	0.12	0.48			25	0.46	0.12	0.23	0.70		
Sample size						6.64	0.01						0.01	0.91
<35	17	0.32	0.12	0.08	0.57			17	0.31	0.16	−0.01	0.62		
≥35	20	0.21	0.08	0.05	0.37			20	0.28	0.12	0.05	0.51		
Intervention duration						8.24	0.03						2.59	0.46
≤ 3 h	6	0.38	0.14	0.09	0.66			6	0.89	0.71	−0.50	2.27		
3 h−2 months	11	0.69	0.32	0.07	1.31			11	0.29	0.12	0.05	0.53		
2– 6 months (including 2 months)	12	0.13	0.08	−0.04	0.29			12	0.00	0.19	−0.37	0.37		
>6 months	8	0.09	0.07	−0.05	0.22			8	0.24	0.08	0.08	0.40		
Number of intervention methods used						6.40	0.04						3.99	0.14
1–3 methods used	3	0.19	0.12	−0.05	0.43			3	0.12	0.17	−0.21	0.44		
4–10 methods used	17	0.49	0.14	0.22	0.75			17	0.55	0.18	0.20	0.90		
>10 methods used	17	0.11	0.06	−0.02	0.23			17	0.16	0.11	−0.06	0.38		
Setting						5.83	0.21						3.21	0.36
School	12	0.08	0.08	−0.06	0.23			12	0.09	0.07	−0.05	0.24		
University	4	0.44	0.30	−0.14	1.03			4	−0.89	0.91	−2.68	0.90		
Lab	4	0.18	0.18	−0.17	0.53			4	0.38	0.16	0.07	0.69		
Community	5	0.49	0.19	0.11	0.87			5	0.53	0.08	0.38	0.68		
Other	12	0.33	0.16	0.02	0.64			12	0.40	0.22	−0.03	0.84		
Physical activity at baseline						6.54	0.09						4.00	0.26
Not meeting guideline	17	0.11	0.07	−0.03	0.26			17	0.23	0.08	0.08	0.38		
Meeting guideline	3	0.69	0.28	0.14	1.24			3	0.62	0.35	−0.07	1.32		
Mixed	9	0.45	0.17	0.11	0.78			9	0.62	0.32	−0.01	1.25		
Unreported	8	0.22	0.15	−0.08	0.52			8	−0.01	0.21	−0.43	0.40		
Positive affective variables measure						0.25	0.88						2.84	0.24
Affect	6	0.25	0.06	0.13	0.37			6	0.23	0.14	−0.05	0.50		
Emotional state	24	0.22	0.09	0.05	0.38			24	0.15	0.10	−0.05	0.34		
Affect and emotional state	7	0.34	0.25	−0.16	0.83			7	0.94	0.47	0.02	1.85		
Physical activity measure						9.11	0.10						5.44	0.36
MVPA (subjective)	9	0.16	0.09	−0.02	0.34			9	0.05	0.19	−0.31	0.42		
Steps	4	0.21	0.09	0.04	0.38			4	0.42	0.23	−0.02	0.86		
Frequency	6	0.84	0.44	−0.03	1.71			6	1.14	0.68	−0.19	2.46		
Intensity	6	0.20	0.13	−0.05	0.45			6	0.18	0.14	−0.09	0.45		
Multiple	10	0.16	0.11	−0.06	0.37			10	0.04	0.08	−0.12	0.20		
Theory						10.32	0.01						14.80	0.02
No framework explicitly mentioned	4	0.39	0.22	−0.04	0.82			4	0.25	0.16	−0.07	0.56		
Social cognitive theory	3	0.01	0.19	−0.37	0.39			3	0.16	0.12	−0.08	0.41		
The transtheoretical model	3	0.27	0.11	0.07	0.48			3	0.38	0.14	0.12	0.65		
Theory of planned behavior	3	0.53	0.45	−0.36	1.41			3	0.27	0.27	−0.26	0.81		
Self–determination theory	3	0.80	0.24	0.33	1.27			3	0.57	0.12	0.33	0.81		
Multiple	16	0.08	0.07	−0.06	0.22			16	0.01	0.12	−0.23	0.25		
Others	5	0.44	0.26	−0.08	0.95			5	1.03	0.43	0.18	1.88		
Study design						0.58	0.45						0.68	0.41
Randomized controlled trial	13	0.38	0.19	0.01	0.75			13	0.42	0.16	0.10	0.74		
Quasi–experimental study	24	0.23	0.07	0.09	0.36			24	0.25	0.11	0.03	0.47		
Study quality						0.57	0.75						4.08	0.13
Low (1–2)	7	0.17	0.20	−0.22	0.57			7	0.02	0.12	−0.22	0.26		
Medium (3–4)	24	0.26	0.08	0.11	0.41			24	0.34	0.13	0.09	0.58		
High (5–6)	6	0.38	0.20	0.00	0.77			6	0.39	0.21	−0.03	0.80		

*Calculated using combined comparisons (Two arms, Control vs. AB, Control vs. ABC) with a total of 37 data sets. Moderator analysis was only done on moderators present in >3 studies*.

**Figure 3:**
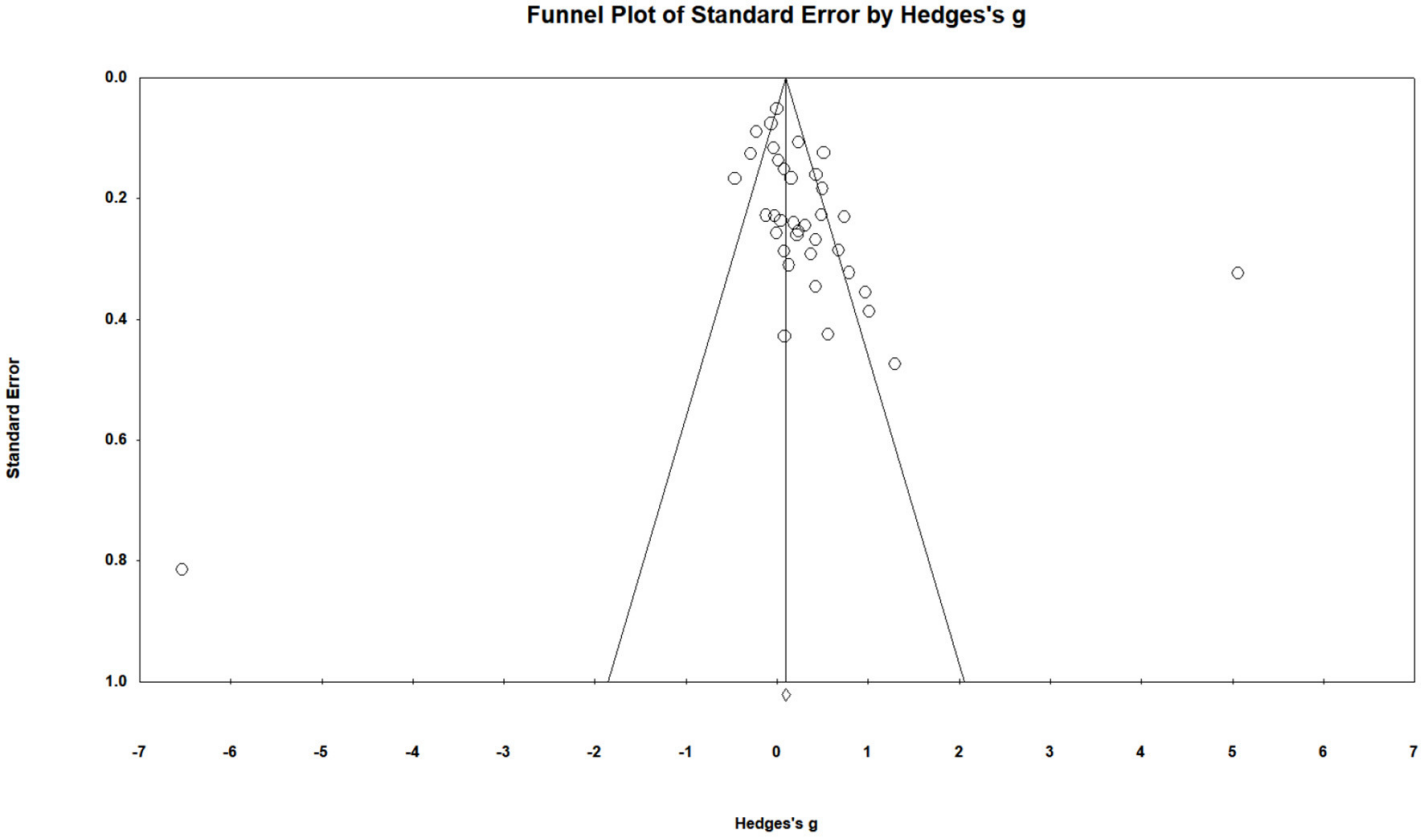
Funnel plot of physical activity in this review.

Next we would report the moderating effects of demographic and methodological factors on PA. The overall effect size of interventions on PA was *g* = 0.30 (95% CI = 0.10–0.48) (see Appendix 7). However, the Egger regression intercept for the PA data was not significant (*t* = 1.84 (35), *p* = 0.07) (see Figure 3). The results of meta-analytic moderation analyses showed that small sample bias was not a significant moderator of PA outcomes (*Q* = 0.01, *p* = 0.91). Age was a significant moderator of PA outcomes (*Q* = 19.23, *p* < 0.01), with a maximum effect size reported for the mean age between 18 and 35 years (*g* = 0.44, 95% CI = −0.10 to 0.97). Gender was also a significant moderating variable (*Q* = 11.53, *p* < 0.01), with the mixed gender sample reporting larger effect size (*g* = 0.46, 95% CI = 0.23–0.70). In addition, theory was also a significant moderator of PA outcomes (*Q* = 14.80, *p* = 0.02), with the lagest effect sizes of “others” (*g* = 1.03, 95% CI = 0.18–1.88).

### Moderating Effect of Contents Applied in the Intervention on PAVs and PA

We performed 29 meta-analytic moderation analyses based on a refined taxonomy of intervention techniques (Michie et al., 2011; see Table 1). It was not sensible to perform moderating analyses for the remaining 11 techniques because fewer than three intervention groups utilized them. Refer to Appendix 5 for details of the intervention techniques used in each intervention group.

The presence of two intervention techniques increased the variations in PAVs. They were “teach to use prompts/cues” (present *g* = 0.73; absent *g* = 0.26, *p* = 0.02) and “facilitate social comparison” (present *g* = 0.98; absent *g* = 0.26, *p* = 0.01). However, the application of two other intervention techniques could reduce the outcomes of PAVs. They were “barrier identification/problem solving” (present *g* = 0.09; absent *g* = 0.45, *p* = 0.01) and “plan social support/social change” (present *g* = 0.19, absent *g* = 0.45, *p* =0.04). None of the other 25 techniques included in the moderator analysis differed significantly in their effect size estimates between the two study groups, irrelevant of whether they included a specified technique or not (see Table 4).

**Table 4:** Comparison between PAVs and PA, according to whether a specific technique is present or absent in the intervention.

**Technique (moderator)**		**PAVs**								**PA**							
		**Present**			**Absent**			**Q**	***p***	**Present**			**Absent**			**Q**	***p***
		***k***	***g***	**SE**	***k***	***g***	**SE**			***k***	***g***	**SE**	***k***	***g***	**SE**		
1	Provide information on consequences of behavior in general	15	0.29	0.11	34	0.32	0.08	0.05	0.83	15	0.54	0.13	34	0.26	0.09	3.45	0.04
3	Provide information about others' approval	9	0.26	0.15	40	0.32	0.07	0.02	0.73	9	0.18	0.16	40	0.39	0.08	1.35	0.25
4	Provide normative information about others' behavior	5	0.43	0.23	44	0.30	0.07	0.28	0.60	5	0.40	0.26	44	0.35	0.08	0.05	0.83
5	Goal setting (behavior)	19	0.29	0.10	30	0.33	0.08	0.12	0.73	19	0.39	0.11	30	0.32	0.10	0.25	0.61
6	Goal setting (outcome)	6	0.24	0.19	43	0.32	0.07	0.15	0.70	6	0.39	0.22	43	0.35	0.08	0.04	0.84
7	Action planning	28	0.20	0.08	21	0.38	0.10	2.21	0.14	28	0.33	0.09	21	0.38	0.12	0.10	0.75
8	Barrier identification/problem solving	18	0.09	0.10	31	0.45	0.08	7.79	0.01	18	0.19	0.11	31	0.46	0.09	3.43	<0.05
9	Set graded tasks	10	0.16	0.13	39	0.36	0.07	1.87	0.17	10	0.35	0.15	39	0.35	0.08	0.00	0.98
10	Prompt review of behavioral goals	13	0.34	0.12	36	0.30	0.07	0.08	0.78	13	0.65	0.14	36	0.25	0.08	5.67	0.06
11	Prompt review of outcome goals	3	0.43	0.23	46	0.30	0.07	0.28	0.60	3	0.40	0.26	46	0.35	0.08	0.05	0.83
16	Prompt self–monitoring of behavior	24	0.24	0.09	25	0.37	0.09	1.12	0.29	24	0.28	0.11	25	0.42	0.10	0.88	0.35
17	Prompt self-monitoring of behavioral outcome	4	0.15	0.20	45	0.33	0.07	0.75	0.39	4	0.36	0.24	45	0.35	0.08	0.00	0.96
19	Provide feedback on performance	20	0.23	0.10	29	0.37	0.08	1.08	0.30	20	0.27	0.11	29	0.41	0.09	0.86	0.35
20	Provide instruction on when and where to perform the behavior	40	0.31	0.07	9	0.31	0.16	0.03	0.99	40	0.34	0.08	9	0.42	0.18	0.15	0.70
21	Provide instruction on how to perform the behavior	41	0.32	0.07	8	0.27	0.18	0.06	0.80	41	0.36	0.08	8	0.32	0.21	0.04	0.84
22	Model/demonstrate the behavior	28	0.28	0.08	21	0.36	0.10	0.29	0.60	28	0.43	0.09	21	0.23	0.12	1.75	0.19
23	Teach to use prompts/cues	6	0.73	0.20	43	0.26	0.06	5.09	0.02	6	1.33	0.23	43	0.25	0.07	19.80	<0.01
24	Environmental restructuring	15	0.29	0.11	34	0.32	0.08	0.04	0.85	15	0.21	0.12	34	0.42	0.09	1.94	0.16
26	Prompt practice	7	0.26	0.16	42	0.32	0.07	0.13	0.72	7	0.18	0.19	42	0.38	0.08	1.02	0.31
27	Use of follow–up prompts	3	0.19	0.29	46	0.32	0.06	0.19	0.66	3	0.46	0.32	46	0.34	0.07	0.12	0.73
28	Facilitate social comparison	4	0.98	0.25	45	0.26	0.06	7.95	0.01	4	0.97	0.28	45	0.30	0.07	5.45	0.02
29	Plan social support/social change	25	0.19	0.08	24	0.45	0.09	4.11	0.04	25	0.41	0.10	24	0.28	0.11	0.86	0.35
33	Prompt self–talk	4	0.04	0.23	45	0.33	0.07	1.53	0.22	4	0.22	0.26	45	0.36	0.08	0.26	0.61
34	Prompt use of imagery	9	0.35	0.17	40	0.31	0.07	0.05	0.82	9	0.10	0.18	40	0.40	0.08	2.27	0.13
35	Relapse prevention/coping planning	3	0.23	0.23	46	0.32	0.07	0.12	0.73	3	0.52	0.27	46	0.33	0.07	0.47	0.49
36	Stress management/emotional training	24	0.26	0.09	25	0.36	0.09	0.66	0.42	24	0.32	0.10	25	0.39	0.10	0.23	0.63
37	Motivational interviewing	6	0.10	0.19	43	0.34	0.07	1.38	0.24	6	0.33	0.22	43	0.36	0.08	0.02	0.90
38	Time management	4	−0.16	0.25	45	0.34	0.07	3.62	0.06	4	0.08	0.27	45	0.37	0.08	1.08	0.30
39	General communication skills training	5	0.05	0.18	44	0.35	0.07	2.54	0.11	5	0.13	0.20	44	0.38	0.08	1.39	0.24

*k indicates the number of intervention groups adopting/not adopting a particular technique. They are calculated using all comparisons (Two arms, Control vs. A, Control vs. B, Control vs. C) with a total of 49 data sets. Moderator analysis was only done on moderators present in >3 intervention groups*.

The presence of three intervention techniques increased the variations in PA. They were “provide information on consequences of behavior in general” (present *g* = 0.54; absent *g* = 0.26, *p* = 0.04), “teach to use prompts/cues” (present *g* = 1.33; absent *g* = 0.25, *p* < 0.01) and “facilitate social comparison” (present *g* = 0.97; absent *g* = 0.3, *p* = 0.02). However, the application of another intervention technique could reduce the outcomes of PA. It was “barrier identification/problem solving” (present *g* = 0.19; absent *g* = 0.46, *p* < 0.05). None of the other 25 techniques included in the moderator analysis differed significantly in their effect size estimates between the two study groups, irrelevant of whether they included a specified technique or not (see Table 4).

## Discussion

This paper intended to provide a nuanced summary of the characteristics of current research methodologies for PAVs and PA interventions, identify intervention techniques that have been used sparingly, and determine the most compelling theories and techniques in recent researches. Therefore, this investigation had two series of objectives. First, summarize experimental studies targeting PAVs in order to change PA and their characteristics (study characteristics, BCT characteristics, PAV characteristics, PA characteristics); second, investigate the moderating effects of methodology, demographics, and BCTs.

### The Characteristics of Demographics and Methodologies

The retrieved studies suggest that 83.78% of the included studies were of moderate or low quality, only about a third were RCTs, and approximately half were small sample studies. Besides, the majority of the retrieved studies were set in schools or universities, and only one study setting was the worksite. Approximately 70% of the studies did not specify subject genders; nearly 30% of the interventions targeted females only, with only one study exclusively targeting male subjects. Approximately 45% of the studies did not report on the subjects' PA level at baseline (“not meeting guideline” or “meeting guideline”), and the role of PAVs for different initial exercise conditions remained to be explored. Besides, PAVs were measured in various formats and dimensions, but no studies explained the differences and commonalities between those different formats and dimensions. Generally, PA consists of three elements: exercise intensity, exercise duration, and exercise frequency. However, only six of the 37 studies used accelerometers, and the others measured only one or two of the three elements of PA (subject's steps, heart rate, instrument usage, or possible time of exercise). Hence, in future studies, the accuracy of PA measurements could be improved further. Finally, eleven of 40 intervention techniques were utilized by <3 intervention groups, and their effectiveness should be explored better in relevant studies.

### Moderating Effect of Methodological and Demographic Variables on PAVs and PA

The differences of effects between intervention and control conditions on both PAVs (*g* = 0.29; 95% CI = 0.15–0.43) and PA (*g* = 0.30; 95% CI = 0.11–0.49) were significant. Due to the studies' non-negligible heterogeneity, these considerable effect sizes should be interpreted with caution. Furthermore, our survey identified the underlying publication bias [Egger's intercept *t* = 1.65 (35), *p* = 0.02] in PAVs context. Given the significant publication bias in PAV, we further detected a larger effect size for small studies. Borenstein et al. (2009) noted that this pattern of larger effect size for small studies might because we retrieved a biased sample of small studies, but it is also possible that the effect size for small studies is larger for entirely unrelated reasons. That is, the presence of a small-study effect (Sterne et al., 2001) in PAVs may contribute to its publication bias. Under these circumstances, we recommend focusing on high-power studies and reducing studies with small samples to obtain more reliable estimates in future meta-analyses. Overall, no significant variations were found across the number of intervention methods used, PA at baseline, measurement employed, study design, or study quality assessment. However, age, gender, intervention duration, and theoretical framework significantly moderated the finding of PA. These findings were briefly discussed below.

a) *Age moderated PAV and PA*. Studies at the age interval between 36 and 50 years reported the maximum effect size (*g* = 0.48) in the PAV context, and subjects age between 18 and 35 reported the maximum effect size (*g* = 0.44) in the PA context. These results were consistent with those described by Lundqvist et al. (2013) and Vieillard and Gilet (2013): on the one hand, aging was associated with the maintenance of positive affect and the reduction of negative affect; on the other hand, a stimulus rating task showed that older adults had a considerably smaller range of responses to emotional stimuli than youngers. Besides, Kang et al. (2009) showed that separating interventions for different age groups was significantly more effective than not separating. Maybe one appropriate intervention strategy for one age group may not be appropriate for another age group. Therefore, we recommend selecting samples of approximately similar ages in a single study and administering higher intensity emotional stimulation to the young population in such interventions.
b) *Gender moderated PA*. Mixed samples (*g* = 0.30) reported larger changes than female samples (*g* = 0.07) in the PAV context, and mixed samples (*g* = 0.46) also reported larger changes than female samples (*g* = −0.08) in the PA context. These findings are difficult to interpret because there are not enough male-only samples to compare to mixed samples. Future studies where participant gender is used as an ex post facto variable within the same design are needed to shed light on this finding.
c) *Intervention duration moderated PAV*. The results suggested that interventions shorter than 2 months showed the most significant effect size (*g* = 0.69). Based on this result, we take a long-term perspective and recommend that exercise intervention strategies should not be monotonous and constant over time but should be adjusted about once every 2 months in order to facilitate PAV growth.
d) *Theory moderated PAV and PA*. Interventions with SDT (*g* = 0.80) had the most significant impact on PAV outcomes, while interventions without a theoretical basis (*g* = 1.03) had the most significant impact on PA. SDT posits that there are two main types of motivation—intrinsic and extrinsic—and that both are powerful forces shaping who we are and how we act. When individuals are motivated by intrinsic motivation, they may feel self-directed and autonomous (Ryan and Deci, 2000). This result is understandable due to the numerous conceptual and content similarities between intrinsic motivation and PAVs (Wienke and Jekauc, 2016). Parallel to the aforementioned, the interventions without theory presented the most significant impact on PA, which may reveal the limited predictive power of current theoretical frameworks. These findings highlighted the importance of developing theory underpinnings of PA prediction and intervention.

### Moderating Effect of Contents Applied in the Intervention on PAVs and PA

We found “teach to use prompts/cues” and “facilitate social comparison” were related to conceivable positive changes in PAV, and “teach to use prompts/cues,” “facilitate social comparison,” and “provides information on consequences of behavior in general” were related to positive changes in PA. These findings were briefly discussed below:

a) “*Teach to use prompts/cues*” *positively moderated PAV and PA*. The concept of “teach to use prompts/cues” is to instruct people to recognize environmental prompts (e.g., mobile phone reminders) that can be used to remind them to enact an intended behavior. This technique is desired as a planned, systematic delivery of cues to prompt people to do cognitive or metacognitive work on emotional arousal and PA to help people establish task-specific routines, automatic responses, or habits in their daily lives that internalize motivational factors and thus contribute to PA levels (Hayamizu, 1997). The TTM researchers note that teaching to use prompts/cues of PA behavior can facilitate individuals' transition from pre-contemplation stage to contemplation stage or even action stage. However, in explaining why this technique works, this theory only emphasizes consciousness-raising and ignoring PAVs' changes. Therefore, future TTM-based PA intervention studies could additionally consider the role of PAVs.
b) “*Facilitate social comparison*” *moderated PAV and PA*. It is not surprising that this technique enhanced both PAVs and PA, as the technique in line with a critical construct of SDT. The SDT assigns a central role to intrinsic motivation, a construct that is typically operationalized by assessing the degree of enjoyment associated with behavioral preferences (Deci and Ryan, 2008). The concept of “facilitate social comparison” is to draw attention to the performance of others to elicit comparisons. According to SDT, individuals have three necessary psychological needs for intrinsic motivation to adopt and adhere to behaviors: the need for competence, the need for relatedness, and autonomy. We speculate that social comparisons enhance the subjects' sense of competition and the likelihood of perceiving their competence (Kwasnicka et al., 2016).
c) “*Provide information on consequences of behavior in general*” *positively moderated PA*. Its purpose is to provide information on the relationship between PA and its possible consequences in general cases, based on epidemiological data. One possible explanation for the positive effect is that the epidemiological data may have facilitated the valuation of PA as healthy, but this could also be a statistical fluke of the results of multiple comparisons, so further research on this topic is recommended.

In contrast, “barrier identification/problem solving” was negatively associated with PAVs and PA change, and “plan social support/social change” was related to an adverse change in PAVs. These findings were briefly discussed below:

a) “*Barrier identification/ problem solving*” *negatively moderated PAV and PA*. Both Koole and Rothermund (2011) and Gyurak et al. (2011) pointed out the difference between explicit (requires conscious and cognitive effort to initiate and monitor) and implicit (operates without the need for conscious supervision) emotion regulation. Gyurak et al. (2011) also noted that although, by definition, implicit emotion regulation is not intentional (i.e., it is not instigated or guided by explicit intentions), some research emphasizes the goal-directed nature of implicit emotion regulation. This aspect of non-intentionality distinguishes the studies of implicit emotion regulation from most studies of explicit emotion regulation because implicit emotion regulation does not require such explicit instruction, so it is more spontaneous than explicit emotional regulation (Koole and Rothermund, 2011). Given that “barrier identification and problem solving” was defined as prompting the person to think about underlying obstacles and identifying methods to overcome them (Michie et al., 2011), we considered it to be a cognitive variable. In other words, we thought this cognitive variable to be an explicit rather than implicit process, which might have hindered PAVs and subsequent PA growth. In addition, as a common intervention technique based on SCT, we might have to consider its impact on environmental modification and also on affective variables. However, it was also possible that barrier identification was not necessarily ineffective, but instead that the technique was ineffective due to an incorrect implementation method.
b) “*Plan social support/ social change*” *negatively moderated PAV*. Although relatively little research has been done on the relationship between this variable and PAVs, the outcome is understandable. Because planning is an activity that requires the activation of an individual's cognitive resources, we consider this variable also to be an explicit rather than implicit process of emotion regulation. Based on the interaction between cognition and emotion (Liu et al., 2009), we speculate that complex cognition hinders the growth of PAVs. In general, social change is also a common intervention or environmental modification technique based on SCT. Future PA intervention studies using social support/social change need to address the impact on PAVs. However, this could also be a statistical fluke of multiple comparisons, and further research on this is recommended.

At present, new theoretical models of PA change have been developed based on the automatic affective valuation option, such as affective–reflective theory (ART; Brand and Ekkekakis, 2018) and the PA adoption and maintenance model (PAAM model; Strobach et al., 2020). However, they have not yet explored which specific BCTs would be helpful for enhancing positive affective evaluations (PAVs) in the healthy population, and this paper might be considered as a preliminary attempt.

### Limitations and Future Research Directions

Although this review followed the Cochrane handbook for systematic reviews of interventions (Higgins et al., 2019b) as normatively as possible, several limitations still exist. First, the included studies were limited by search terms and language, and it was not possible to include all relevant studies. Future studies should consider including more languages to explore whether there are differences in manipulating positive affect variables and PA across countries or cultural contexts (e.g., Eastern and Western cultural contexts). Second, this study did not include unpublished data. However, given Bellefontaine and Lee (2014) explored the impact of including gray literature and found no significant differences in effect size and methodological quality with or without the inclusion of unpublished studies, we also considered this to be a minor limitation. Third, since it was not possible to split positive and negative affective variables into two, we only excluded negative affective variables. Fourth, due to data limitation, we could not analyze all 40 behavior change techniques listed in Michie et al. (2011), and only 29 BCTs were analyzed. Therefore, rigorous experimental testing using a factorial design that isolates and combines unique techniques is needed. Fifth, this paper focused on exploring the effectiveness of different BCTs, but not the effectiveness of affective change techniques, so more work needs to be done to gain insight into them. Sixth, given the broad age spectrum of the current study population, we expect future studies to narrow their age spectrum to explore age-specific intervention techniques. Seventh, the results might be inflated due to potential unit-of-analysis errors that might exist by using the current analytical methods. According to Cheung (2019) and Higgins et al. (2019a), multi-level meta-analysis and network meta-analysis are probably the best to deal with meta-analysis studies which include several effects from one study. Future studies should consider using them to achieve rigorous estimations.

## Conclusion

Overall, the primary objective was to summarize the demographic, methodological, and BCTs of each study to review gaps in past experimental designs. Descriptive statistics showed that: at least 11 behavior change techniques were rarely used in included studies; the measurements of PAV dimensions and methods were highly inconsistent across studies; accelerometers were still not widely used in PA measurement. Inferential statistics yielded that: age, gender, intervention duration, and theoretical basis had significant moderating effects on PAV or PA outcomes; the utilization of “teach to use prompts/cues,” “facilitate social comparison,” and “provide information on consequences of behavior in general” had positive effects on PA or PAV outcomes; the utilization of “barrier identification/problem solving” and “plan social support/social change” negatively affected on PA or PAV outcomes. However, there was considerable heterogeneity in the findings, and the moderator analyses suggested that these effects may be exaggerated by publication and small sample bias. Nonetheless, this paper has considerable implications for future relative intervention studies, and these findings will serve as a base for future such intervention studies.

## Data Availability Statement

The original contributions presented in the study are included in the article/Supplementary Material, further inquiries can be directed to the corresponding author/s.

## Author Contributions

DJ and CC contributed to the study's conception and design. DJ supervised the entire process. CC organized the database, performed the statistical analysis, and wrote the manuscript. EF and AK supported CC in data extraction and data analysis phases. DJ, EF, and CC contributed to manuscript revision. All authors read and approved the submitted version.

## Conflict of Interest

The authors declare that the research was conducted in the absence of any commercial or financial relationships that could be construed as a potential conflict of interest.
