# Supplementary material for: What Intervention Techniques Are Effective in Changing Positive Affective Variables and Physical Activity? A Systematic Review and Meta-Analysis
Source: Front Psychol. 2021 Jun 10;12:628993. doi: 10.3389/fpsyg.2021.628993 (PMC8222822; doi:10.3389/fpsyg.2021.628993)
Supplement: Supplementary file 1 [file Table_1.docx]

**Supplementary Materials**

**Appendix 1**

*Quality assessment scoring by study in meta-analytic analyses*

|  | Study | | Item 1 | Item 2 | Item 3 | Item 4 | Item 5 | Item 6 | QA score |
| --- | --- | --- | --- | --- | --- | --- | --- | --- | --- |
| 1 | Berg et al., 2020 | | 0 | 0 | 1 | 1 | 0 | 0 | 2 |
|  |  | |  |  |  |  |  |  |  |
| 2 | Taylor, 2020 | | 1 | 0 | 1 | 1 | 0 | 0 | 3 |
|  |  | |  |  |  |  |  |  |  |
| 3 | Invernizzi et al., 2019 | | 0 | 1 | 1 | 1 | 1 | 0 | 4 |
|  |  | |  |  |  |  |  |  |  |
| 4 | Pearce et al., 2019 | | 1 | 1 | 1 | 0 | 0 | 0 | 3 |
|  |  | |  |  |  |  |  |  |  |
| 5 | Rhodes et al., 2019 | | 0 | 0 | 0 | 1 | 1 | 0 | 2 |
|  |  | |  |  |  |  |  |  |  |
| 6 | Robbins et al., 2019 | | 1 | 0 | 1 | 1 | 1 | 0 | 4 |
|  |  | |  |  |  |  |  |  |  |
| 7 | Rodríguez et al., 2019 | | 1 | 0 | 1 | 0 | 1 | 0 | 3 |
|  |  | |  |  |  |  |  |  |  |
| 8 | Vazou et al., 2019 | | 0 | 0 | 1 | 1 | 1 | 0 | 3 |
|  |  | |  |  |  |  |  |  |  |
| 9 | Vitali, et al., 2019 | | 1 | 0 | 1 | 1 | 0 | 0 | 3 |
|  |  | |  |  |  |  |  |  |  |
| 10 | Faro et al., 2019 | | 1 | 1 | 1 | 1 | 0 | 0 | 4 |
|  |  | |  |  |  |  |  |  |  |
| 11 | Gråstén et al., 2019 | | 0 | 0 | 1 | 1 | 1 | 0 | 3 |
|  |  | |  |  |  |  |  |  |  |
| 12 | Andruschko et al., 2018 | | 0 | 0 | 1 | 0 | 0 | 0 | 1 |
|  |  | |  |  |  |  |  |  |  |
| 13 | Miragall et al., 2018 | | 1 | 0 | 1 | 0 | 1 | 1 | 4 |
|  |  | |  |  |  |  |  |  |  |
| 14 | Hutchinson et al., 2018 | | 1 | 0 | 1 | 1 | 1 | 0 | 4 |
|  |  | |  |  |  |  |  |  |  |
| 15 | Noradechanuntet al., 2017 | | 1 | 1 | 1 | 1 | 1 | 0 | 5 |
|  |  | |  |  |  |  |  |  |  |
| 16 | Niedermeier et al., 2017 | | 0 | 1 | 1 | 1 | 1 | 1 | 5 |
|  |  | |  |  |  |  |  |  |  |
| 17 | Billing, 2017 | | 0 | 1 | 0 | 1 | 0 | 0 | 2 |
|  |  | |  |  |  |  |  |  |  |
| 18 | Wang et al., 2015 | | 0 | 0 | 1 | 1 | 0 | 1 | 3 |
|  |  | |  |  |  |  |  |  |  |
| 19 | Kraft et al., 2015 | | 1 | 0 | 1 | 0 | 0 | 1 | 3 |
|  |  | |  |  |  |  |  |  |  |
| 20 | Jekauc, 2015 | | 0 | 0 | 0 | 1 | 1 | 0 | 2 |
|  |  | |  |  |  |  |  |  |  |
| 21 | Mark et al., 2013 | | 1 | 1 | 1 | 1 | 1 | 1 | 6 |
|  |  | |  |  |  |  |  |  |  |
| 22 | Focht, 2013 | | 1 | 0 | 1 | 1 | 1 | 0 | 4 |
|  |  | |  |  |  |  |  |  |  |
| 23 | Fitzsimons et al., 2012 | | 0 | 0 | 1 | 1 | 1 | 1 | 4 |
|  |  | |  |  |  |  |  |  |  |
| 24 | Schneider et al., 2011 | | 0 | 0 | 1 | 1 | 1 | 0 | 3 |
|  |  | |  |  |  |  |  |  |  |
| 25 | Louise et al., 2010 | | 0 | 0 | 1 | 1 | 0 | 1 | 3 |
|  |  | |  |  |  |  |  |  |  |
| 26 | Rhodes, Warburton, & Bredin, 2009 | | 1 | 1 | 1 | 1 | 0 | 1 | 5 |
|  |  | |  |  |  |  |  |  |  |
| 27 | Focht, 2009 | | 0 | 0 | 1 | 1 | 1 | 0 | 3 |
|  |  | |  |  |  |  |  |  |  |
| 28 | Edmunds et al., 2008 | | 0 | 0 | 0 | 1 | 1 | 0 | 2 |
|  |  | |  |  |  |  |  |  |  |
| 29 | Annesi et al., 2008 | | 0 | 0 | 1 | 1 | 1 | 0 | 3 |
|  |  | |  |  |  |  |  |  |  |
| 30 | Baker et al., 2008 | | 0 | 1 | 1 | 1 | 1 | 1 | 5 |
|  |  | |  |  |  |  |  |  |  |
| 31 | Focht et al., 2007 | | 1 | 0 | 1 | 1 | 0 | 0 | 3 |
|  |  | |  |  |  |  |  |  |  |
| 32 | Rose et al., 2007 | | 0 | 0 | 1 | 1 | 1 | 0 | 3 |
|  |  | |  |  |  |  |  |  |  |
| 33 | Robbins et al., 2006 | | 0 | 0 | 1 | 1 | 0 | 1 | 3 |
|  |  | |  |  |  |  |  |  |  |
| 34 | Jamner et al., 2004 | | 1 | 0 | 1 | 1 | 1 | 0 | 4 |
|  |  | |  |  |  |  |  |  |  |
| 35 | McAuley et al., 2003 | | 1 | 0 | 0 | 0 | 1 | 1 | 3 |
|  |  | |  |  |  |  |  |  |  |
| 36 | Digelidis et al., 2003 | | 0 | 0 | 0 | 1 | 0 | 0 | 1 |
|  |  |  |  |  |  |  |  |  |  |
| 37 | Nichols et al., 2000 | | 1 | 1 | 1 | 1 | 0 | 1 | 5 |

*Note.* Item 1 = Did the study describe the participant eligibility criteria?; Item 2 = Were the participants randomly selected (or for experimental studies, was the process of randomization clearly described and adequately carried out?); Item 3 = Did the study report the sources and details of physical activity assessment and did the instruments have acceptable reliability for the specific age group?; Item 4 = Did the study report the sources and details of assessment of potential correlates and did all of the methods have acceptable reliability?; Item 5 = Did the study report a power calculation and was the study adequately powered to detect hypothesized relationships?; Item 6 = Did the study report the numbers of individuals who completed each of the different measures and did participants complete at least 80% of physical activity measures?; QA = quality assessment; 1-2 = low, 3-4 = medium, 5-6 = high

**Appendix 2**

*General Study Characteristics*

|  | Study | |  | Participants in Intervention Group | | | |  | Intervention | |  | Affective dimensions | |  | PA | |
| --- | --- | --- | --- | --- | --- | --- | --- | --- | --- | --- | --- | --- | --- | --- | --- | --- |
|  | Primary Author &  Year | Setting |  | PA Level | N | Female % | M_age_ (SD) |  | Theory | Length |  | Type | Measure |  | Type | Measure |
| 1 | Berg et al., 2020 | Internet |  | Unreported | 226 | 96.02 | 27±6.68 |  | SDT, DMP | 4 weeks |  | Positive affects | PANAS-X |  | PA | PATCQ |
|  |  |  |  |  |  |  |  |  |  |  |  |  |  |  |  |  |
| 2 | Taylor, 2020 | Internet& university |  | Unreported | 19 | 100 | College freshman |  | SCT, SDT | 9 weeks |  | Enjoyment | PACES |  | PA | IPAQ |
|  |  |  |  |  |  |  |  |  |  |  |  |  |  |  |  |  |
| 3 | Invernizzi et al., 2019 | School |  | Mixed | 62 | 46.77 | 10.5±0.5 |  | CPT | 12 weeks |  | Enjoyment | PACES |  | PA | PAQ-C |
|  |  |  |  |  |  |  |  |  |  |  |  |  |  |  |  |  |
| 4 | Pearce et al., 2019 | School & home |  | Unreported | 63 | 64 | 8 to 13 |  | SMT, SCT, PMT, TTM, TPB | 8 months |  | Enjoyment | PACES |  | MVPA | Accelerometer, PAQ-C |
|  |  |  |  |  |  |  |  |  |  |  |  |  |  |  |  |  |
| 5 | Rhodes et al., 2019 | Family |  | Not meeting guideline | 73 | Null | 11.5±1.3 |  | TPB, SDT | 13 weeks |  | Affective attitude | Semantic differentia |  | Equipment usage | Exercise log |
|  |  |  |  |  |  |  |  |  |  |  |  |  |  |  |  |  |
| 6 | Robbins et al., 2019 | School & internet |  | Mixed | 1519 | 100 | 12.05±1.01 |  | HPM, SDT | 17 weeks |  | Enjoyment | PACES |  | MVPA | Accelerometer |
|  |  |  |  |  |  |  |  |  |  |  |  |  |  |  |  |  |
| 7 | Rodríguez et al., 2019 | School |  | Mixed | 131 | 51.91 | 8.66±1.77 |  | TGM | 8 weeks |  | Affective valence | FS |  | PA | Pedometer |
|  |  |  |  |  |  |  |  |  |  |  |  |  |  |  |  |  |
| 8 | Vazou et al., 2019 | School |  | Mixed | 148 | 52% | 10.39±0.98 |  | ART | 30 minutes |  | Enjoyment, affective valence | S-PACES, FS |  | PA | Accelerometer |
|  |  |  |  |  |  |  |  |  |  |  |  |  |  |  |  |  |
| 9 | Vitali, et al., 2019 | School |  | Mixed | 80 | 48.75 | 10.45±0.23 |  | Null | 4 years |  | Enjoyment | PACES |  | PA | CLASS |
|  |  |  |  |  |  |  |  |  |  |  |  |  |  |  |  |  |
| 10 | Faro et al., 2019 | University |  | Not meeting guideline | 34 | 100 | 27.3±4.5 |  | DMM | 4 weeks |  | Enjoyment, affect | PACES, FS |  | HR | HRM |
|  |  |  |  |  |  |  |  |  |  |  |  |  |  |  |  |  |
| 11 | Gråstén et al., 2019 | School |  | Mixed | 661 | 52.60 | 12.12±0.33 |  | AGT, SEM | 2 years |  | Enjoyment | PEES |  | MVPA | HBSC, Actigraph |
|  |  |  |  |  |  |  |  |  |  |  |  |  |  |  |  |  |
| 12 | Andruschko et al., 2018 | School |  | Not meeting guideline | 20 | 100 | 13.2±0.9 |  | SCT | 6 months |  | Enjoyment | Likert scale |  | PA, MVPA | Accelerometer |
|  |  |  |  |  |  |  |  |  |  |  |  |  |  |  |  |  |
| 13 | Miragall et al., 2018 | Internet & university |  | Not meeting guideline | 76 | 85.5 | 22.18±3.71 |  | TTM | 3 weeks |  | Enjoyment | PACES |  | PA | Pedometer |
|  |  |  |  |  |  |  |  |  |  |  |  |  |  |  |  |  |
| 14 | Hutchinson et al., 2018 | Lab |  | Meeting Guideline | 17 | 47.1 | 28.1±9.9 |  | HT, DMM | 48 hours |  | Affective valence, Remembered pleasure | FS, VAS |  | HR | HRM |
|  |  |  |  |  |  |  |  |  |  |  |  |  |  |  |  |  |
| 15 | Noradechanunt et al., 2017 | Community |  | Not meeting guideline | 39 | 74.36 | 66.6±6.7 |  | Null | 12 weeks |  | Enjoyment | PACES |  | PA | PASE |
|  |  |  |  |  |  |  |  |  |  |  |  |  |  |  |  |  |
| 16 | Niedermeier et al., 2017 | Outdoor, lab |  | Mixed | 42 | 48 | 32.00±11.90 |  | DMM, CM | 170 minutes |  | Mood states | FS, MSS |  | HR | HRM |
|  |  |  |  |  |  |  |  |  |  |  |  |  |  |  |  |  |
| 17 | Billing, 2017 | Telephone |  | Not meeting guideline | 40 | 90 | 39±12 |  | DMM, HT, SCT | 12 weeks |  | Affect, enjoyment | FS, PACES |  | MVPA | Accelerometer, 7DPAR |
|  |  |  |  |  |  |  |  |  |  |  |  |  |  |  |  |  |
| 18 | Wang et al., 2015 | School |  | Unreported | 62 | 50 | 22.3±1.51 |  | SDT, SNST | 8 weeks |  | Enjoyment | IMI |  | PA | IPAQ |
|  |  |  |  |  |  |  |  |  |  |  |  |  |  |  |  |  |
| 19 | Kraft et al., 2015 | University |  | Mixed | 20 | 50 | 22.06±3.6 |  | Null | 15minutes*3 |  | Enjoyment | VAS |  | HR, MET | HRM, Accelerometer |
|  |  |  |  |  |  |  |  |  |  |  |  |  |  |  |  |  |
| 20 | Jekauc, 2015 | Community |  | Unreported | 41 | 87.8 | 46.12 |  | SDT | 8 weeks |  | Enjoyment | PACES |  | Exercise Adherence | Attendance lists |
|  |  |  |  |  |  |  |  |  |  |  |  |  |  |  |  |  |
| 21 | Mark et al., 2013 | Family |  | Not meeting guideline | 30 families | 50.84 | 36.83±6.30 |  | TPB | 6 weeks |  | affective attitude | Likert scale |  | leisure-time PA | GLTEQ |
|  |  |  |  |  |  |  |  |  |  |  |  |  |  |  |  |  |
| 22 | Focht, 2013 | Lab |  | Not meeting guideline | 23 | 100 | 26.62±5.16 |  | SCT, TPB | 30mins/ 10mins |  | Affective valence, exercise-induced feeling | FS, EFI |  | PA | LTEQ |
|  |  |  |  |  |  |  |  |  |  |  |  |  |  |  |  |  |
| 23 | Fitzsimons et al., 2012 | Community |  | Not meeting guideline | 79 | 88.73 | 49±9 |  | TTM | 48 weeks |  | Affect | PANAS |  | PA | Pedometer |
|  |  |  |  |  |  |  |  |  |  |  |  |  |  |  |  |  |
| 24 | Schneider et al., 2011 | School |  | Not meeting guideline | 122 | 100 | 15.04±0.78 |  | SDT | 9 months |  | Enjoyment | PACES |  | PA | 3DPAR |
|  |  |  |  |  |  |  |  |  |  |  |  |  |  |  |  |  |
| 25 | Louise et al., 2010 | School |  | Unreported | 221 | 59.28 | 13.29±0.99 |  | SMT | 16 weeks |  | Enjoyment | PACES |  | LTPA | 7DPAR |
|  |  |  |  |  |  |  |  |  |  |  |  |  |  |  |  |  |
| 26 | Rhodes, Warburton, & Bredin, 2009 | University |  | Meeting guideline | 29 | 0 | 22.7±4.0 |  | TPB | 6 weeks |  | Affective attitude | Semantic differential |  | Adherence to exercise | Attendance list |
|  |  |  |  |  |  |  |  |  |  |  |  |  |  |  |  |  |
| 27 | Focht, 2009 | Lab & Outdoor |  | Meeting guideline | 35 | 100 | 22.14±1.73 |  | TPB | 10 minutes |  | Affective valence, enjoyment, exercise-induced feelings | FS, EFI, SES |  | PA, HR | LTEQ, HRM |
|  |  |  |  |  |  |  |  |  |  |  |  |  |  |  |  |  |
| 28 | Edmunds et al., 2008 | University |  | Mixed | 56 | 100 | 21.32±5.56 |  | SDT | 10 weeks |  | Affect | PANAS |  | Exercise Behavior | Attendance list |
|  |  |  |  |  |  |  |  |  |  |  |  |  |  |  |  |  |
| 29 | Annesi et al., 2008 | Community |  | Unreported | 269 | 59 | 10.6±1.1 |  | SET, SCT | 1 year |  | Vigor | POMS |  | Voluntary Physical Activity | SSMVPA |
|  |  |  |  |  |  |  |  |  |  |  |  |  |  |  |  |  |
| 30 | Baker et al., 2008 | Community |  | Not meeting guideline | 79 | 79.75 | 49.2±8.9 |  | TTM | 12 weeks |  | Affect | PANAS |  | PA | Pedometer |
|  |  |  |  |  |  |  |  |  |  |  |  |  |  |  |  |  |
| 31 | Focht et al., 2007 | Lab |  | Not meeting guideline | 18 | 55.56 | 24.10±3.40 |  | SCT | 8 weeks |  | Affective valence, exercise-induced feelings | FS, EFI |  | - | - |
|  |  |  |  |  |  |  |  |  |  |  |  |  |  |  |  |  |
| 32 | Rose et al., 2007 | Lab |  | Not meeting guideline | 19 | 100 | 39.37±10.29 |  | DMM, SCT, SET | 20 minutes |  | Affective valence | FS |  | HR | HRM |
|  |  |  |  |  |  |  |  |  |  |  |  |  |  |  |  |  |
| 33 | Robbins et al., 2006 | School |  | Not meeting guideline | 77 | 100 | 12.13±0.91 |  | HPM, TTM, SCT | 12 weeks |  | Enjoyment | PACES |  | PA | CAAL |
|  |  |  |  |  |  |  |  |  |  |  |  |  |  |  |  |  |
| 34 | Jamner et al., 2004 | School |  | Not meeting guideline | 58 | 100 | 14.94±0.79 |  | Null | 4 months |  | Enjoyment | PACES |  | PA | 2DPAR, SUPAS |
|  |  |  |  |  |  |  |  |  |  |  |  |  |  |  |  |  |
| 35 | McAuley et al., 2003 | Gymnasium |  | Not meeting guideline | 174 | 71.84 | 65.5 |  | SCT | 6 months |  | Exercise affect | FS |  | Exercise frequency | Exercise log |
|  |  |  |  |  |  |  |  |  |  |  |  |  |  |  |  |  |
| 36 | Digelidis et al., 2003 | School |  | Unreported | 782 | 52.17 | 12.05±0.73 |  | TPB, GPT, TARGETM | 1 year |  | Enjoyment | IMI |  | Exercise behavior | EFS |
|  |  |  |  |  |  |  |  |  |  |  |  |  |  |  |  |  |
| 37 | Nichols et al., 2000 | Worksite |  | Not meeting guideline | 160 | 78.13 | 42.0±9.7 |  | SCT, TTM | 33 months |  | Enjoyment | PACES |  | PA | 7DPAR |

*Note.* PATCQ = Physical activity time consuming questionnaire; PAQ-C = The physical activity questionnaire for children; CLASS = The children’s leisure activities study survey; 7DPAR = 7-day physical activity recall; 3DPAR = 3-day physical Activity Recall; 2DPAR = 2-day physical activity recall; HRM = Heart rate monitor; HBSC = The health behavior in School-aged children research protocol; PASE = The physical activity scale for the elderly; IPAQ = The short-form of the international physical activity questionnaire; GLTEQ = A modified Godin leisure-time exercise questionnaire; LTEQ = Leisure-time exercise questionnaire; SSMVPA = A single-item scale to assess the moderate to vigorous physical activity over the previous week; CAAL = the child and adolescent activity log; SUPAS = the Stanford usual physical activity scale; EFS = 6-point exercise frequency scale; PACES = The physical activity enjoyment scale; S-PACES = Shorted physical activity enjoyment scale for children; FS = The feeling scale; PEES = The PE enjoyment scale; DMP = Dualistic Model of Passion; VAS = Visual analog scale; MSS = A mood survey scale; IMI = The intrinsic motivation inventory; EFI = The Exercise-induced Feeling Inventory; PANAS = The positive and negative affect schedule; PANAS-X = The positive and negative affect schedule-expanded form; TCMM = The trans-contextual model of motivation;SES = Single-item enjoyment scale; POMS = The tension and vigor scales of the profile of mood states-short Form; CPT = Challenge point theory; DMM = The dual-mode model; HPM = the health promotion model; SMT = Self-management theory; PMT = Protection motivation theory; SDT = Self-determination Theory; TGM = Tactical games model; ART = Affective reflective theory; AGT = Achievement goal theory; SEM = Social ecological model; SCT = Social cognitive theory; TTM = The transtheoretical model; TPB = Theory of planned behavior; CM = The circumplex model; SNST = Social network site theory; SMT = Social marketing theory; SET = Self-efficacy theory; GPT = Goal perspectives theory; TARGETM = The TARGET model

**Appendix 3**

*Constructs, dimensions, and measurements of positive affective variables in the included studies*

| Constructs | Measurement | Dimensions | Studies |
| --- | --- | --- | --- |
| Affect | | | |
|  | FS | Affective valence | 7, 8, 10, 14, 17, 22, 27, 31, 32, 35 |
|  | PANAS | Positive affect | 1, 23, 28, 30 |
|  | | | |
| Emotional state | | | |
| Enjoyment | PACES | PAE | 2, 3, 4, 8, 9, 10, 6, 13, 16, 17, 20, 24, 25, 33, 34, 37 |
|  |  |  |  |
|  | PEES | PEE | 11 |
|  |  |  |  |
|  | VAS | Enjoyment | 19 |
|  |  |  |  |
|  | IMI | Enjoyment | 18, 36 |
|  |  |  |  |
|  | SES | Enjoyment | 12, 27 |
|  |  |  |  |
|  |  |  |  |
| Pleasure | VAS | Remembered pleasure | 14 |
|  | | | |
| Exercise-induced feelings | EFI | Revitalization | 22, 27, 31 |
|  |  | Positive engagement | 22, 27, 31 |
|  |  |  |  |
| Affective attitude | SD | Affective attitude | 5, 26 |
|  |  |  |  |
|  | Likert scale | Affective attitude | 21 |
|  |  |  |  |
| Mood states | POMS | Vigor | 29 |
|  | | | |
|  | MSS | Activation | 16 |
|  |  | Excitement | 16 |

*Note.* Study content in Appendix 6; FS= Feeling scale; PANAS = Positive and negative affect schedule; PEES = The PE enjoyment scale; SES = Single-item enjoyment scale; VAS = Visual analog scale; IMI = Intrinsic motivation inventory; PACES = The physical activity enjoyment scale; PAE = Physical activity enjoyment; PEE = The PE enjoyment; EFI = Exercise-induced feeling inventory; SD= Semantic differential items on seven-point scales; POMS = Profile of mood states; MSS = Mood survey scale

**Appendix 4**

*Physical activity assessment methods and measurements in each study*

| Assessment methods | Variables | Measurements | Studies |
| --- | --- | --- | --- |
| Objective methods | | | |
|  | | | |
| Pedometer | Steps | The Omron HJ-109E Step-O-Meter | 23, 30 |
|  |  | Pedometer Fitbit One | 13 |
|  |  | Yamax Digiwalker SW-650 (Yamax Corporation, Toyko, Japan) | 7 |
|  |  |  |  |
| Accelerometer | MVPA | ActiGraph(did not state the type, manufacturer, and place of origin) | 17 |
|  |  | ActiGraph GT3X+ (ActiGraph, Ft. Walton Beach, FL, USA) | 4, 6 |
|  |  |  |  |
|  | MVPA, PA | Actigraph Model 7164 (Fort Walton Beach, FL, USA) | 12 |
|  |  |  |  |
|  | MET | ActiGraph GT3X+ (Pensacola, FL) | 19 |
|  |  | The SenseWear Armband Monitor (BodyMedia, Pittsburgh PA) | 8 |
|  |  |  |  |
| Log or list | Equipment usage | Log | 5, 35 |
|  |  |  |  |
|  | Exercise Adherence | Attendance lists | 20, 26, 28 |
|  |  |  |  |
|  |  |  |  |
| HR monitoring | HR or %Max HR or HR at VT | HR monitor | 10, 14, 16, 19, 27, 32 |
|  |  |  |  |
|  |  |  |  |
| Subjective methods | | | |
|  | | | |
| Questionnaires | MVPA | 7DPAR | 12 |
|  |  | IPAQ | 2, 18 |
|  |  | HBSC | 11 |
|  |  | 3DPAR | 24 |
|  |  | PAQ-C | 4 |
|  | LTPA | 7DPAR | 25 |
|  |  | LTEQ | 22 |
|  |  |  |  |
|  | VPA | SSMVPA | 29 |
|  |  |  |  |
|  | PA | 7DPAR | 37 |
|  |  | 2DPAR | 34 |
|  |  | PASE | 15 |
|  |  | CAAL | 33 |
|  |  | PAQ-C  CLASS | 3  9 |
|  |  | PATCQ | 1 |
|  |  |  |  |
|  | LTPA | GLTEQ | 21 |
|  |  |  |  |
|  | Lifestyle activity | SUPAS | 34 |
|  |  |  |  |
|  | Exercise behavior | EFS | 36 |

*Note.* Study content in Appendix 10; MVPA = Moderate to vigorous physical activity; PA = Physical activity; LTPA = Leisure-time physical activity; LTEQ = Leisure-time exercise questionnaire; GLTEQ = A modified Godin leisure-time exercise questionnaire; SSMVPA = A single-item scale to assess the moderate to vigorous physical activity over the previous week; VPA = Voluntary physical activity; HR = Heart rate; MET = The metabolic equivalent of task; 7DPAR = 7-day physical activity recall; 3DPAR = 3-day physical activity recall; 2DPAR = 2-day physical activity recall; IPAQ = The short-form of the international physical activity questionnaire; PATCQ = Physical activity time consuming questionnaire; HBSC = The health behavior in school-aged children; PASE = The physical activity scale for the elderly; CAAL = the child and adolescent activity log; PAQ-C = The physical activity questionnaire for older children; CLASS = The children’s leisure activities study survey; SUPAS = The Stanford usual physical activity scale; EFS = 6-point exercise frequency scale

**Appendix 5**

*Intervention techniques included in each intervention group in current review*

| Studies | | Intervention techniques |
| --- | --- | --- |
| 1 | Berg et al., 2020 | 16, 34, 37 |
| 2 | Taylor, 2020 | 5, 8, 10, 16, 19, 20, 23, 29, 34, 36, 38 |
| 3 | Invernizzi et al., 2019 | 1, 5, 7, 8, 9, 16, 19, 20, 21, 22, 28, 29, 36 |
| 4 | Pearce et al., 2019 | 1, 5, 7, 8, 9, 20, 21, 22, 36 |
| 5 | Rhodes et al., 2019 | 5, 7, 8, 10, 16, 20, 21, 24,29, 34 |
| 6 | Rodríguez et al., 2019 | 10, 20, 21, 22, 26 |
| 7 | Vazou et al., 2019 | 3, 7, 13, 16, 19, 20, 21, 22 |
| 8 | Vitali, et al., 2019 | 1, 5, 10, 20, 21, 22, 23, 29 |
| 9 | Faro et al., 2019 | 16, 20, 21, 22 |
| 10 | Robbins et al., 2019 | 2, 8, 16, 19, 29, 36, 37 |
| 11 | Gråstén et al., 2019 |  |
|  | Group 1 | 7, 8, 16, 19, 20, 21, 22, 24, 26, 29, 36, 39 |
|  | Group 2 | 7, 8, 16, 19, 20, 21, 22, 24, 26, 29, 36, 39 |
| 12 | Andruschko et al., 2018 | 5, 7, 8, 16, 19, 20, 21, 22, 29, 36, 37, 38 |
| 13 | Miragall et al., 2018 |  |
|  | Group 1 | 1, 5, 6, 16, 19, 36 |
|  | Group 2 | 1, 5, 6, 36 |
| 14 | Hutchinson et al., 2018 | 10, 16, 20, 21, 24, 36 |
| 15 | Noradechanuntet al., 2017 |  |
|  | Group 1 | 7, 20, 21, 22, 27 |
|  | Group 2 | 7, 20, 21, 22, 27 |
| 16 | Niedermeier et al., 2017 |  |
|  | Group 1 | 5, 9, 20, 21, 24, 29 |
|  | Group 2 | 5, 9, 20, 21, 24, 29 |
| 17 | Billing, 2017 | 5, 6, 7, 10, 12, 16, 23, 27, 29, 34, 36 |
| 18 | Wang et al., 2015 |  |
|  | Group 1 | 7, 20, 21, 22 |
|  | Group 2 | 1, 3, 4, 7, 20, 21, 22, 23, 25, 28, 29, 36 |
|  | Group 3 | 1, 3, 4, 8, 16, 21, 22, 23, 24, 25, 28, 29, 36 |
| 19 | Kraft et al., 2015 |  |
|  | Group 1 | 20, 21, 24, 34 |
|  | Group 2 | 20, 21, 24, 26, 34 |
| 20 | Jekauc, 2015 | 7,10,11,18,19,20,21, 22, 28,36 |
| 21 | Mark et al., 2013 | 3, 16, 20, 21, 24, 34 |
| 22 | Focht, 2013 | 9, 20 |
| 23 | Fitzsimons et al., 2012 |  |
|  | Group 1 | 7, 8, 9, 10, 16, 17, 19, 21, 22, 29, 35, 36, 37 |
|  | Group 2 | 7, 8, 9, 10, 16, 17, 19, 21, 22, 29, 35, 36, 37 |
| 24 | Schneider et al., 2011 |  |
|  | Group 1 | 1, 3, 5, 7, 8, 16, 19, 20, 21, 22, 29, 36 |
|  | Group 2 | 1, 3, 5, 7, 8, 16, 19, 20, 21, 22, 29, 36 |
| 25 | Louise et al., 2010 | 1, 8, 10, 19, 20, 21, 22, 24, 29, 36, 39 |
| 26 | Rhodes, Warburton, & Bredin, 2009 | 7, 16, 20, 21, 24, 34 |
| 27 | Focht, 2009 | 20, 24 |
| 28 | Edmunds et al., 2008 | 4, 6, 7, 10, 11, 19, 20, 21, 22, 26, 29, 33 |
| 29 | Annesi et al., 2008 | 1, 4, 5, 6, 7, 10, 11, 19, 20, 21, 22, 26, 29, 33 |
| 30 | Baker et al., 2008 | 7, 8, 9, 10, 16, 17, 19, 21, 22, 29, 35, 36, 37 |
| 31 | Focht et al., 2007 |  |
|  | Group 1 | 7, 20, 21 |
|  | Group 2 | 7, 20, 21 |
| 32 | Rose et al., 2007 |  |
|  | Group 1 | 5, 20, 21, 36 |
|  | Group 2 | 5, 20, 21 |
|  | Group 3 | 5, 20, 21 |
| 33 | Robbins et al., 2006 | 1, 2, 3, 4, 7, 8, 19, 20, 21, 22, 24, 29, 36 |
| 34 | Jamner et al., 2004 | 1, 3, 5, 7, 8, 16, 19, 20, 21, 22, 29, 36 |
| 35 | McAuley et al., 2003 | 7, 9, 20, 21, 22 |
| 36 | Digelidis et al., 2003 | 1, 5, 6, 7, 9, 17, 19, 20, 21, 22, 29, 33, 34, 36, 38, 39 |
| 37 | Nichols et al., 2000 | 1, 3, 5, 7, 8, 16, 18, 20, 21, 22, 23, 24, 26, 29, 33, 38, 39 |

*Note*. Intervention technique content in **Table 1**.

**Appendix 6**

**
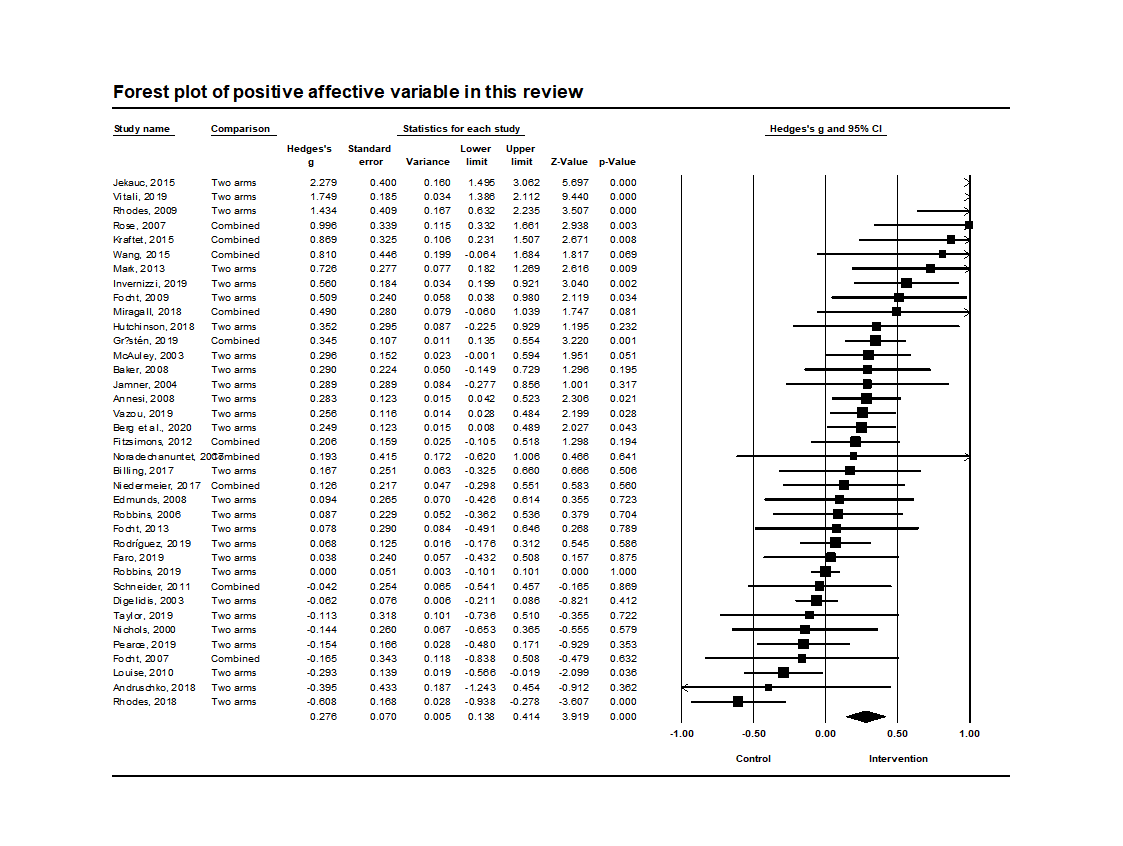
**

**Appendix 7**

**
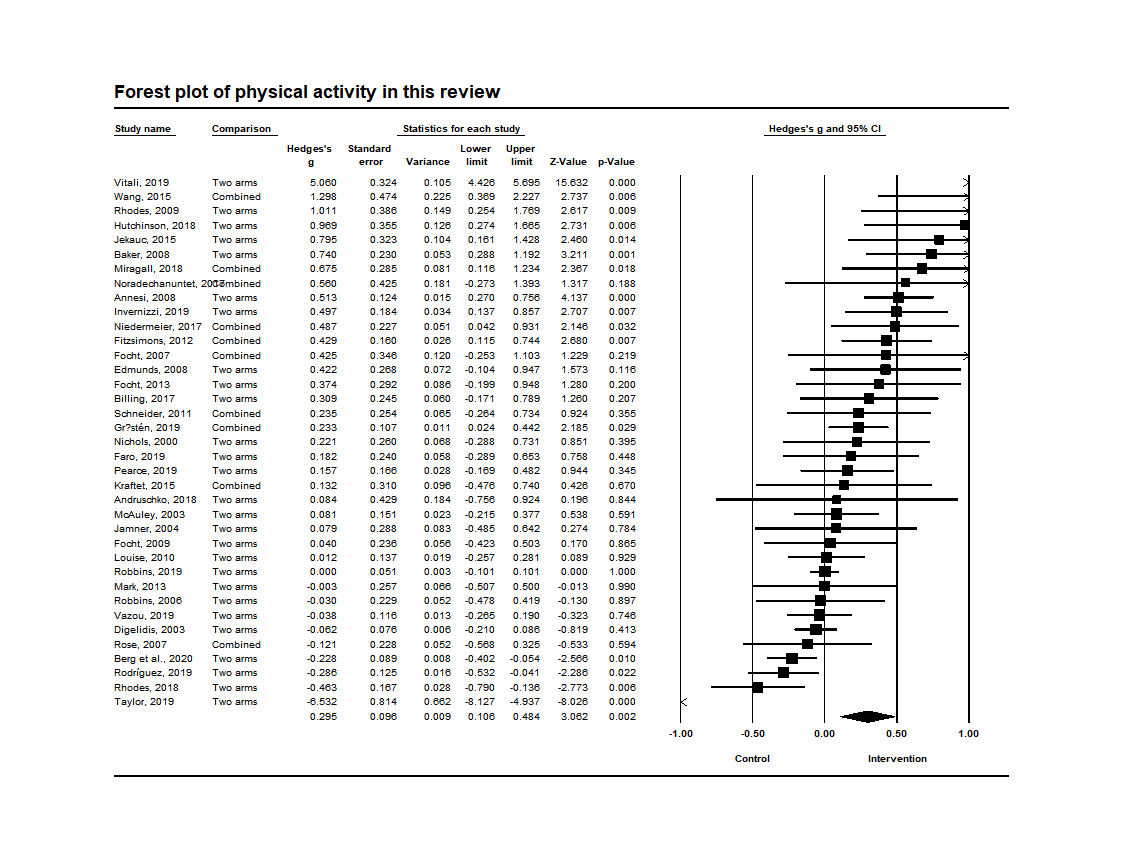
****Appendix 8**

*References for Articles included in meta-analytic analyses*

1. Berg, S., Forest, J., & Stenseng, F. (2020). When Passion Does Not Change, but Emotions Do: Testing a Social Media Intervention Related to Exercise Activity Engagement. *Frontiers in Psychology*, 11, 71.
2. Taylor, M. S. (2020). Fresh start: A group-based intervention to promote physical activity among college freshman (Doctoral dissertation).
3. Invernizzi PL, Crotti M, Bosio A, Cavaggioni L, Alberti G, Scurati R. Multi-teaching styles approach and active reflection: Effectiveness in improving fitness level, motor competence, enjoyment, amount of physical activity, and effects on the perception of physical education lessons in primary school children. *Sustainability*. 2019 Jan;11(2):405.
4. Pearce K, Dollman J. Healthy for Life Pilot Study: A Multicomponent School and Home Based Physical Activity Intervention for Disadvantaged Children. *International journal of environmental research and public health*. 2019 Jan;16(16):2935.
5. Rhodes RE, Beauchamp MR, Blanchard CM, Bredin SS, Warburton DE, Maddison R. Predictors of stationary cycling exergame use among inactive children in the family home. *Psychology of Sport and Exercise*. 2019 Mar 1;41:181-90.
6. Robbins LB, Wen F, Ling J. Mediators of Physical Activity Behavior Change in the “Girls on the Move” Intervention. *Nursing research*. 2019 Jul 1;68(4):257-66.
7. Rodríguez-Negro J, Yanci J. Which instructional models influence more on perceived exertion, affective valence, physical activity level, and class time in physical education?. *Educational Psychology*. 2019 May 9:1-4.
8. Vazou S, Mischo A, Ladwig MA, Ekkekakis P, Welk G. Psychologically informed physical fitness practice in schools: A field experiment. *Psychology of Sport and Exercise*. 2019 Jan 1;40:143-51.
9. Vitali F, Robazza C, Bortoli L, Bertinato L, Schena F, Lanza M. Enhancing fitness, enjoyment, and physical self-efficacy in primary school children: a DEDIPAC naturalistic study. *PeerJ*. 2019 Feb 20;7:e6436.
10. Faro J, Wright JA, Hayman LL, Hastie M, Gona PN, Whiteley JA. Functional resistance training and affective response in female college-age students. *Medicine & Science in Sports & Exercise*. 2019 Jun 1;51(6):1186-94.
11. Gråstén A, Yli‐Piipari S. The Patterns of Moderate to Vigorous Physical Activity and Physical Education Enjoyment Through a 2‐Year School‐Based Program. *Journal of School Health*. 2019 Feb;89(2):88-98.
12. Andruschko J, Okely AD, Pearson P. A school-based physical activity and motor development program for low-fit adolescent females: the Sport4Fun pilot randomized controlled trial. *Journal of Motor Learning and Development*. 2018 Dec 1;6(2):345-56.
13. Miragall M, Domínguez-Rodríguez A, Navarro J, Cebolla A, Baños RM. Increasing physical activity through an Internet-based motivational intervention supported by pedometers in a sample of sedentary students: A randomised controlled trial. *Psychology & health*. 2018 Apr 3;33(4):465-82.
14. Hutchinson JC, Jones L, Vitti SN, Moore A, Dalton PC, O'Neil BJ. The influence of self-selected music on affect-regulated exercise intensity and remembered pleasure during treadmill running. *Sport, Exercise, and Performance Psychology*. 2018 Feb;7(1):80.
15. Noradechanunt C, Worsley A, Groeller H. Thai Yoga improves physical function and well-being in older adults: A randomised controlled trial. *Journal of science and medicine in sport*. 2017 May 1;20(5):494-501.
16. Niedermeier M, Einwanger J, Hartl A, Kopp M. Affective responses in mountain hiking—A randomized crossover trial focusing on differences between indoor and outdoor activity. *PLoS One*. 2017;12(5).
17. Billing L. The Efficacy of Affective Behavioral Strategies for Increasing Physical Activity: Implications for Harnessing the Dual-Mode Model.
18. Wang JC, Leng HK, Kee YH. Use of Facebook in physical activity intervention programme: Test of self-determination theory.
19. Kraft JA, Russell WD, Clark N, Helm J, Jackson A. Influence of experience level on physical activity during interactive video gaming. *Journal of Physical Activity and Health*. 2015 Jun 1;12(6):794-800.
20. Jekauc D. Enjoyment during exercise mediates the effects of an intervention on exercise adherence. *Psychology*. 2015 Jan 13;6(01):48.
21. Mark RS, Rhodes RE. Testing the effectiveness of exercise videogame bikes among families in the home-setting: a pilot study. *Journal of Physical Activity and Health*. 2013 Feb 1;10(2):211-21.
22. Focht BC. Affective responses to 10-minute and 30-minute walks in sedentary, overweight women: Relationships with theory-based correlates of walking for exercise. *Psychology of Sport and Exercise*. 2013 Sep 1;14(5):759-66.
23. Fitzsimons CF, Baker G, Gray SR, Nimmo MA, Mutrie N. Does physical activity counselling enhance the effects of a pedometer-based intervention over the long-term: 12-month findings from the Walking for Wellbeing in the west study. *BMC public health*. 2012 Dec;12(1):206.
24. Schneider M, Cooper DM. Enjoyment of exercise moderates the impact of a school-based physical activity intervention. *International Journal of Behavioral Nutrition and Physical Activity*. 2011 Dec;8(1):64.
25. Louise Bush P, Laberge S, Laforest S. Physical activity promotion among underserved adolescents:“make it fun, easy, and popular”. *Health Promotion Practice*. 2010 May;11(3_suppl):79S-87S.
26. Rhodes RE, Warburton DE, Bredin SS. Predicting the effect of interactive video bikes on exercise adherence: An efficacy trial. *Psychology, health & medicine*. 2009 Dec 1;14(6):631-40.
27. Focht BC. Brief walks in outdoor and laboratory environments: effects on affective responses, enjoyment, and intentions to walk for exercise. *Research quarterly for exercise and sport*. 2009 Sep 1;80(3):611-20.
28. Edmunds J, Ntoumanis N, Duda JL. Testing a self‐determination theory‐based teaching style intervention in the exercise domain. *European journal of social psychology*. 2008 Mar;38(2):375-88.
29. Annesi JJ, Faigenbaum AD, Westcott WL, Smith AE. Relations of self-appraisal and mood changes with voluntary physical activity changes in African American preadolescents in an after-school care intervention. *Journal of sports science & medicine*. 2008 Jun;7(2):260.
30. Baker G, Gray SR, Wright A, Fitzsimons C, Nimmo M, Lowry R, Mutrie N, Scottish Physical Activity Research Collaboration. The effect of a pedometer-based community walking intervention" Walking for Wellbeing in the West" on physical activity levels and health outcomes: a 12-week randomized controlled trial. *International Journal of Behavioral Nutrition and Physical Activity*. 2008 Dec 1;5(1):44.
31. Focht BC, Knapp DJ, Gavin TP, Raedeke TD, Hickner RC. Affective and self-efficacy responses to acute aerobic exercise in sedentary older and younger adults. *Journal of Aging and Physical Activity*. 2007 Apr 1;15(2):123-38.
32. Rose EA, Parfitt G. A quantitative analysis and qualitative explanation of the individual differences in affective responses to prescribed and self-selected exercise intensities. *Journal of Sport and Exercise Psychology*. 2007 Jun 1;29(3):281-309.
33. Robbins LB, Gretebeck KA, Kazanis AS, Pender NJ. Girls on the move program to increase physical activity participation. *Nursing research*. 2006 May 1;55(3):206-16.
34. Jamner MS, Spruijt-Metz D, Bassin S, Cooper DM. A controlled evaluation of a school-based intervention to promote physical activity among sedentary adolescent females: project FAB. *Journal of adolescent health*. 2004 Apr 1;34(4):279-89.
35. McAuley E, Jerome GJ, Marquez DX, Elavsky S, Blissmer B. Exercise self-efficacy in older adults: social, affective, and behavioral influences. *Annals of Behavioral Medicine*. 2003 Jan 1;25(1):1.
36. Digelidis N, Papaioannou A, Laparidis K, Christodoulidis T. A one-year intervention in 7th grade physical education classes aiming to change motivational climate and attitudes towards exercise. *Psychology of Sport and exercise*. 2003 Jul 1;4(3):195-210.
37. Nichols JF, Wellman E, Caparosa S, Sallis JF, Calfas KJ, Rowe R. Impact of a worksite behavioral skills intervention. A*merican Journal of Health Promotion*. 2000 Mar;14(4):218-21.
